# Improving pain, function and quality of life in end-stage knee osteoarthritis: a patient-preference cohort study on whole-body vibration and exercise as bridging therapies for total knee replacement

**DOI:** 10.1186/s42836-025-00301-6

**Published:** 2025-03-10

**Authors:** Kendrew Yu-Hei Choi, Wai-Wang Chau, Linda Man-Kuen Li, Sammie Yuk-Lam Ng, Boie Po-Yee Lo, Michael Tim-Yun Ong, Patrick Shu-Hang Yung

**Affiliations:** 1 Department of Orthopaedics and Traumatology, Prince of Wales Hospital, Hong Kong SAR, China; 2 Department of Orthopaedics and Traumatology, Chinese University of Hong Kong, Hong Kong SAR, China; 3 Department of Physiotherapy, Prince of Wales Hospital, Hong Kong SAR, China

**Keywords:** Exercise, Whole body vibration, End-stage knee osteoarthritis

## Abstract

**Background:**

End-stage knee osteoarthritis (OA) patients awaiting total knee replacement (TKR) often experience prolonged wait times and worsening symptoms. Whole-body vibration (WBV) has shown potential benefits in OA management. This study compared the efficacy of supervised exercise therapy alone and combined with WBV in reducing pain and improving function in this population.

**Methods:**

In this prospective cohort study, 555 patients with end-stage knee OA awaiting TKR were allocated to three groups: Exercise (*n* = 227), Exercise + WBV (*n* = 127), and Control (*n* = 201). The Exercise and Exercise + WBV groups underwent an 8-week intervention comprising strength and flexibility exercises. Primary outcomes were pain (Numeric Pain Rating Scale, NPRS) and function (Knee Injury and Osteoarthritis Outcome Score, KOOS). Outcomes were assessed at baseline and after the final treatment session. Statistical analysis included ANOVA with *post-hoc* Bonferroni correction for baseline comparisons and paired *t*-tests for longitudinal comparisons. Minimal Clinically Important Difference (MCID) and Minimal Detectable Change (MDC) were calculated to assess the clinical significance of the results.

**Results:**

Both exercise modalities significantly reduced knee pain from baseline to final session (*P* < 0.001). The Exercise + WBV group showed a larger reduction in NPRS score (mean ± standard deviation (SD); from 5.57 ± 1.82 to 4.65 ± 2.15) compared to the Exercise group (from 5.35 ± 2.11 to 4.88 ± 1.96), exceeding both MCID (0.94 vs. 0.45) and MDC (0.34 vs. 0.27) thresholds. The Exercise + WBV group demonstrated significant improvements in KOOS subscales (KOOS-KP: 54.31 ± 16.95 to 60.04 ± 17.13, *P* < 0.001; KOOS-S: 57.27 ± 19.56 to 60.50 ± 18.07, *P* = 0.033; KOOS-ADL: 66.99 ± 19.42 to 71.52 ± 16.32, *P* = 0.003), while the Exercise group did not. These improvements in KOOS subscales met or exceeded the MDC (ranging from 2.42 to 3.99) but showed variable clinical significance relative to MCID (− 0.49 to 0.04). The Exercise + WBV group also showed significant improvement in knee ROM (110.68° ± 16.52° to 115.43° ± 18.59°, *P* < 0.001), while the Exercise group did not.

**Conclusion:**

Both interventions effectively reduced pain and improved function in end-stage knee OA patients awaiting TKR, with the addition of WBV leading to greater improvements in several outcomes. Particularly in pain reduction, changes exceeded both MCID and MDC thresholds, suggesting WBV’s potential as a clinically valuable adjunct to exercise therapy. While some improvements in functional outcomes were statistically significant and surpassed MDC values, their clinical significance varied, future research should focus on optimizing WBV protocols and investigating long-term effects to guide clinical practice in managing patients awaiting TKR.

**Supplementary Information:**

The online version contains supplementary material available at 10.1186/s42836-025-00301-6.

## Background

Knee osteoarthritis (OA) is a leading cause of disability worldwide, with a global prevalence of 3.8% in females and 2.8% in males, respectively [1]. The increasing prevalence of knee OA, driven in part by an aging population, has led to a growing demand for total knee replacement (TKR) surgery [2]. However, this demand has resulted in long waits for surgery in many countries, sometimes exceeding 48 months [3–5]. During these extended waits, OA symptoms such as pain and stiffness continue to significantly impact patients’ quality of life [6, 7].

Conservative management options, including exercise and whole-body vibration (WBV), may help alleviate symptoms and improve function in patients awaiting TKR. Exercise therapy has been shown to reduce pain [8, 9] and improve physical function [9] in patients with early to moderate knee OA. However, its effects on end-stage knee OA and TKR candidates have not been extensively studied [10, 11]. The severe joint damage and pain associated with end-stage knee OA may limit the effectiveness of exercise therapy and increase the risk of exacerbating symptoms [12].

WBV [13, 14] has emerged as a promising adjunct to exercise therapy for knee OA [15, 16]. WBV is safe to use [17] and is thought to reduce knee OA pain [18], improve strength [19] and enhance functional performance [20, 21] by stimulating neuromuscular function, proprioception, and joint stability [22, 23]. However, the effects of WBV have not been investigated in end-stage knee OA patients awaiting TKR [24–26]. Like exercise therapy, end-stage knee OA may also limit the efficacy of WBV.

Combining exercise therapy with WBV may offer synergistic benefits for end-stage knee OA patients due to the targeted neuromuscular stimulation and joint stability benefits from vibration alongside conditioning from exercise [19, 22, 27]. Studying such approaches specifically in end-stage knee OA patients awaiting joint replacement is crucial given the rising demand, prolonged wait times, and need for conservative bridging therapies [12, 28].

Despite the promising results of WBV in earlier stages of OA, its effect, when added to exercise in end-stage knee OA, awaiting TKR remain unknown. Addressing this gap is critical considering the expanding need for effective solutions during surgical delays. This was a prospective cohort study to compare the efficacy of supervised exercise therapy alone and combined with WBV in reducing pain and improving physical function among end-stage knee OA patients awaiting TKR. We hypothesized that the combination of exercise and WBV would confer greater improvements compared to exercise alone. The primary objective was to assess changes in knee pain, and self-reported physical function; while secondary objectives included evaluating changes in objective functional performance, and quality of life.

## Materials and methods

### Study design and ethics

This was a prospective cohort study conducted to compare the efficacy of supervised exercise therapy alone and in combination with whole-body vibration among patients with end-stage knee OA awaiting TKR. It was approved by The Joint Chinese University of Hong Kong—New Territories East Cluster Clinical Research Ethics Committee (Reference No.: 2020.401). All participants provided written informed consent, and participant confidentiality was ensured throughout the study. This study was registered at clinicaltrials.gov with registration number NCT06183177.

### Participants

**Fig. 1:**
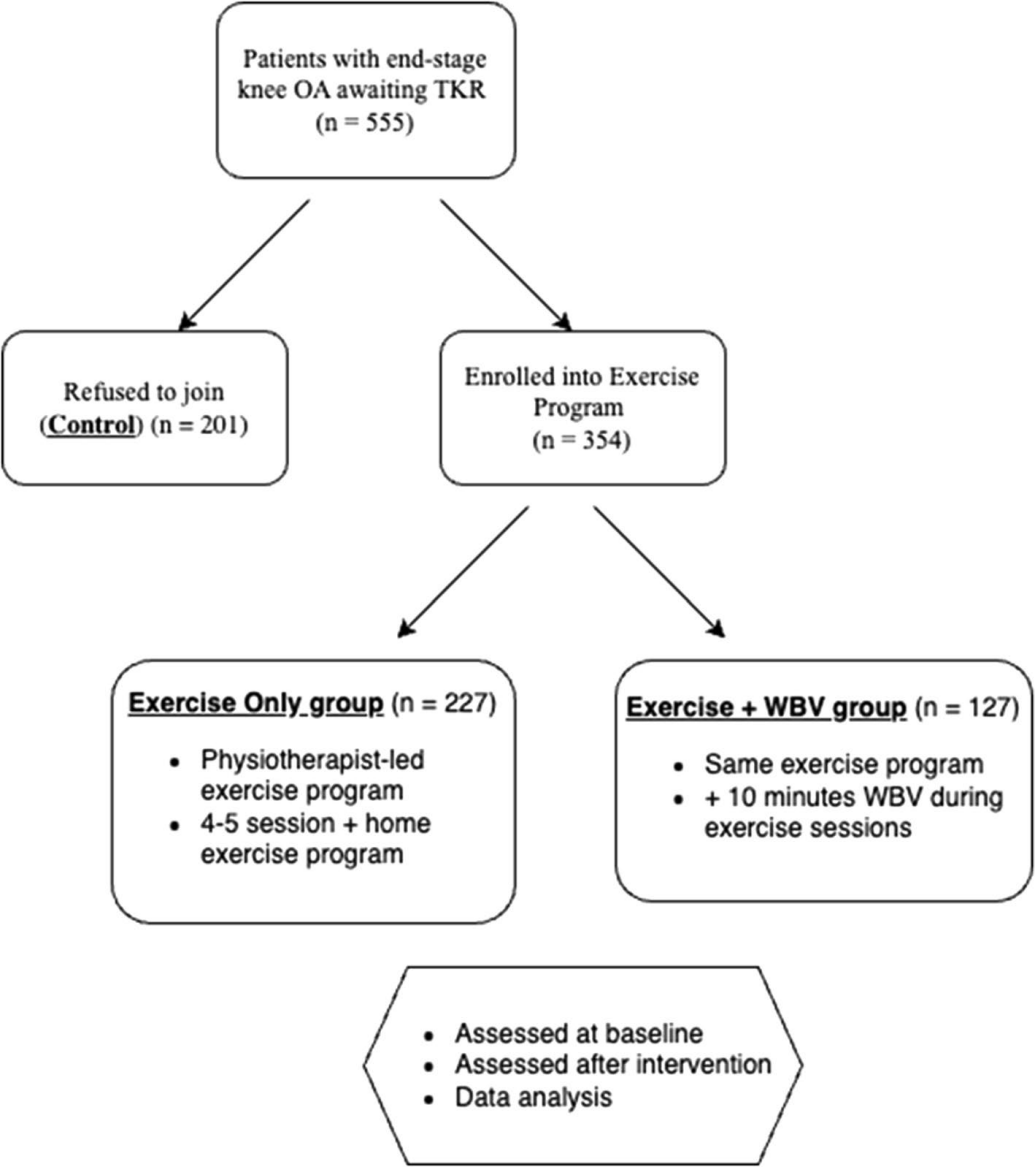
Flow diagram of study design

Patients with end-stage knee OA, awaiting primary TKR were recruited from Prince of Wales Hospital, Hong Kong between October 2021, and January 2023 (Fig. 1).

### Inclusion and exclusion criteria

TKR selection followed the EULAR guidelines, including those participants with severe pain, significant functional limitation and radiographic evidence of joint damage [29]. Exclusion criteria included: severe physical conditions (e.g., uncontrolled cardiovascular disease, severe neurological disorders) or psychological conditions (e.g., severe depression, cognitive impairment) that would hinder participation (Table 1).

**Table 1:** Exclusion Criteria for all participants

Exclusion Criteria Category	Specific Conditions or Factors
Cardiovascular conditions	◦ Metal/synthetic/electrical implants (e.g., pacemaker, artificial cardiac valves, recent stents) ◦ Cardiovascular diseases (e.g., acute thromboses, increased thrombotic afflictions in recent 6 months)
Implant/devices	◦ Implants (e.g., hip, knee or other joint replacements)
Medical conditions	◦ Respiratory diseases ◦ Abdominal diseases (e.g., gallstones) ◦ Urological diseases (e.g., kidney and bladder stones) ◦ Gynecological diseases with intrauterine devices ◦ Neurological diseases (e.g., epilepsy within the last 2 years, spasticity) ◦ Acute injuries to the head ◦ Severe osteoporosis ◦ Tumors with metastases in the musculoskeletal system
Patient characteristics	◦ Body mass exceeding the maximum loaded weight of the whole body vibration machine ◦ Pregnancy

These criteria were assessed through questionnaire, physical examination and review of medical records

A total of 555 patients were enrolled and allocated to one of three groups: “Exercise group” (*n* = 227), “Exercise + Whole-Body Vibration (WBV) group” (*n* = 127), or “Control group” (*n* = 201) following convenience sampling method without randomization. This study employed a patient-preference design to assess efficiency, which was deemed appropriate for the research objective. The rationale for this allocation method was to ensure patient adherence and to reflect real-world clinical practice, where patient preferences often guide treatment decisions.

### Interventions

The interventions period lasted 8 weeks, with participants in the exercise and exercise + WBV group attending 4–5 sessions. Each session consisted of a 20-min education talk, 30 min of group exercises, and 30 min of individual exercise. Handouts, videos, and activity diaries were provided as supplemental resources.

Exercise group (*n* = 227).

**Fig. 2:**
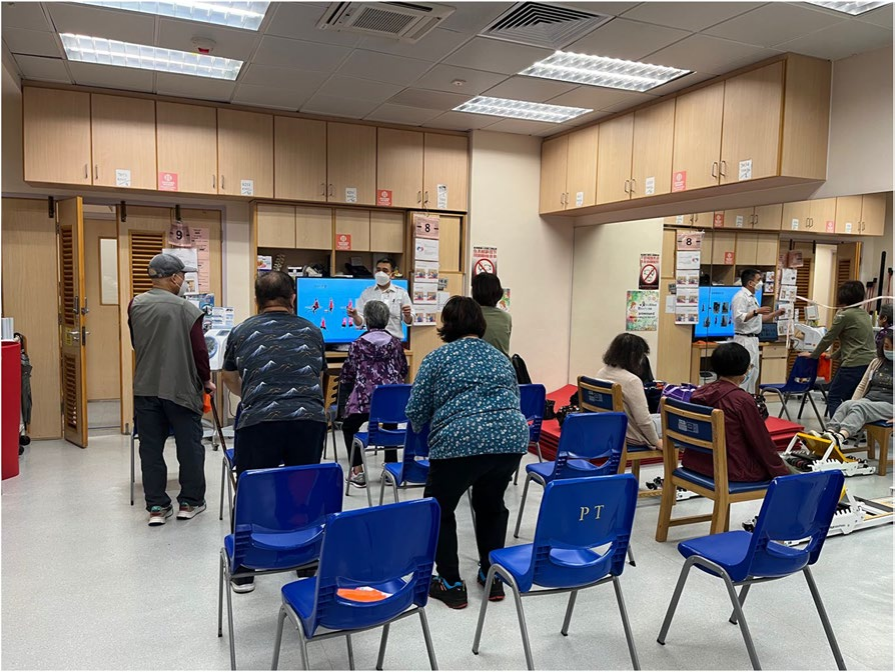
Participants were attending individual/group exercise sessions

The exercise program was led by licensed physiotherapists and included with education, strengthening, stretching, functional, and balance exercises (Fig. 2). Sessions comprised:

- ◦ 20 minutes of knee OA education (Table 2) [30–32].
- ◦ 60-minute strengthening exercises for hips, quadriceps, hamstrings, calves; stretching; functional exercises; balance/proprioception exercises performed in group and individually.

**Fig. 3:**
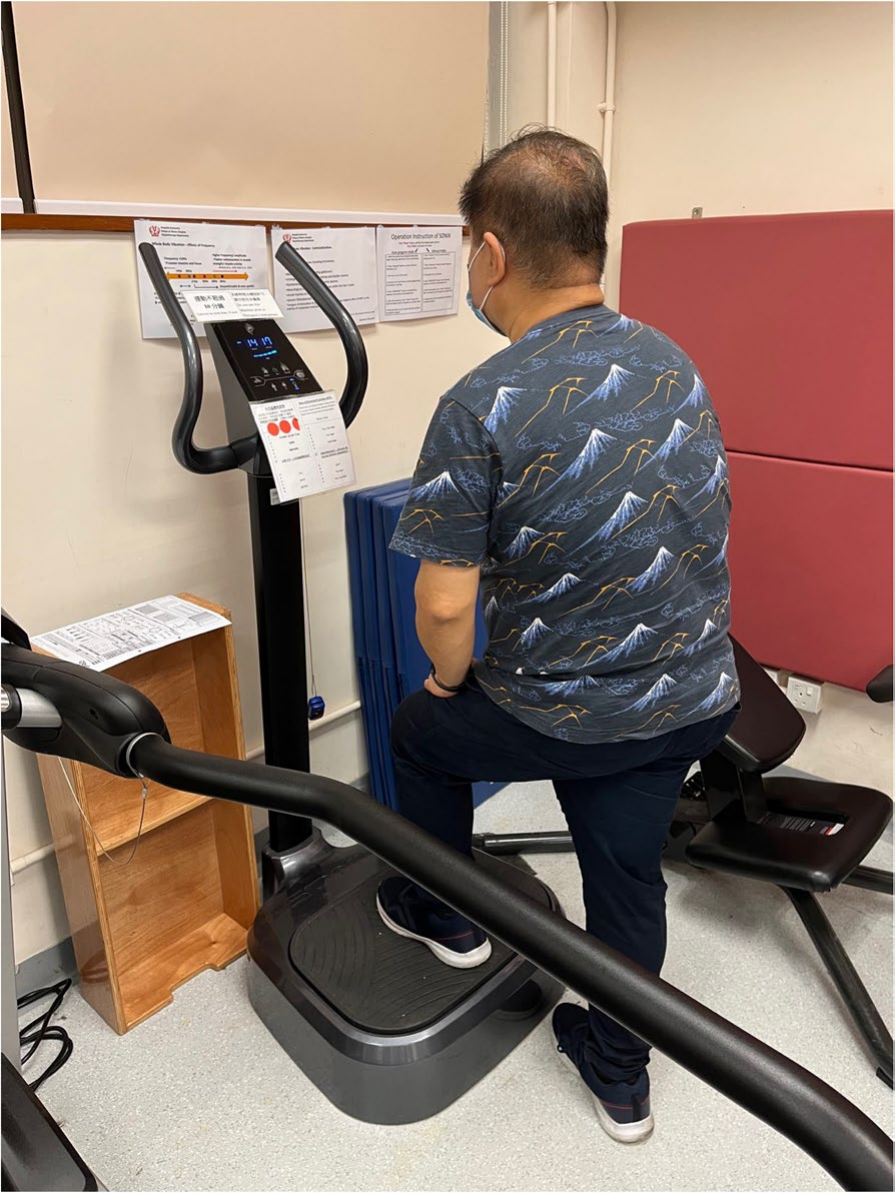
Forward lunges for hip flexor strengthening using the WBV machine

The exercises included the following:

- ◦ Semi-squatting, standing hamstrings curl, standing with hip knee flexed in 90 degrees and calf raises (2–3 sets of 10–15 repetitions). (Fig. 3)
- ◦ Hip abduction, adduction, flexion, extension (2–3 sets of 10–15 repetitions).
- ◦ Resisted knee extension, hip abduction/flexion and ankle plantar flexion with TheraBand in sitting (2–3 sets of 10–15 repetitions).
- ◦ Stretching: Hamstrings, quadriceps, and gastrocnemius (10 repetitions of 30 s holds).
- ◦ Balance exercises: Single leg stands, toe raises (2–3 sets of 30–60 s).
- ◦ Intensity progressively increased from 1–2 lbs to 3–5 lbs weights.
- ◦ Duration increased from 30 s holds/10 reps to 45–60 s holds/15–20 reps.
- ◦ Exercises were modified according to ability/tolerance and aimed to strengthen and stabilize the knee and improve flexibility and proprioception.

Participants were encouraged to perform same set of exercise in their home exercise program, their compliance was monitored through exercise logs and follow-up calls.

Exercise + WBV group (*n* = 127).

**Table 2:**
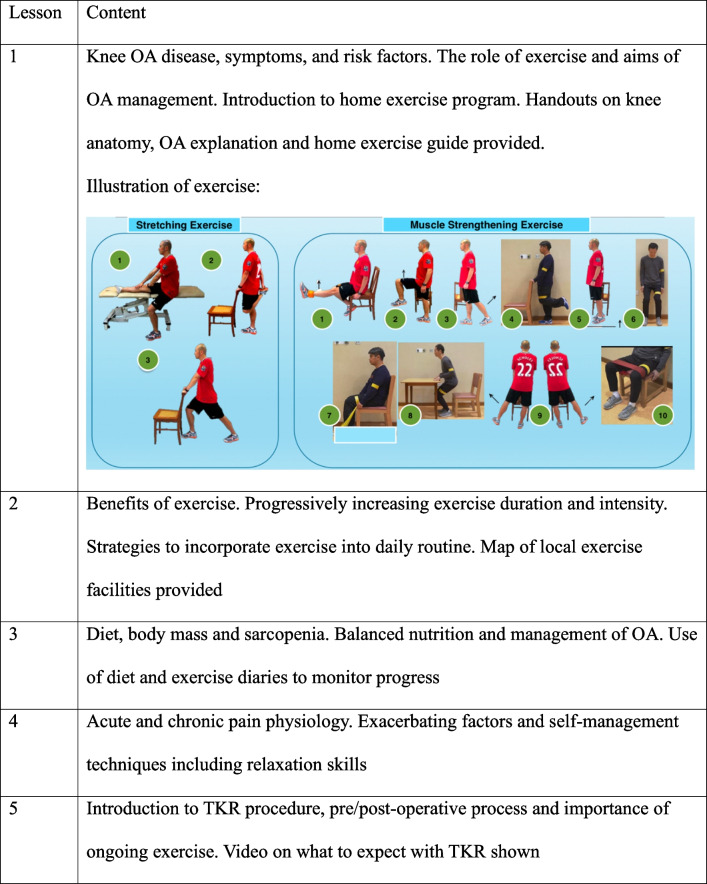
Topics included in Educational Theory Classe

**Fig. 4:**
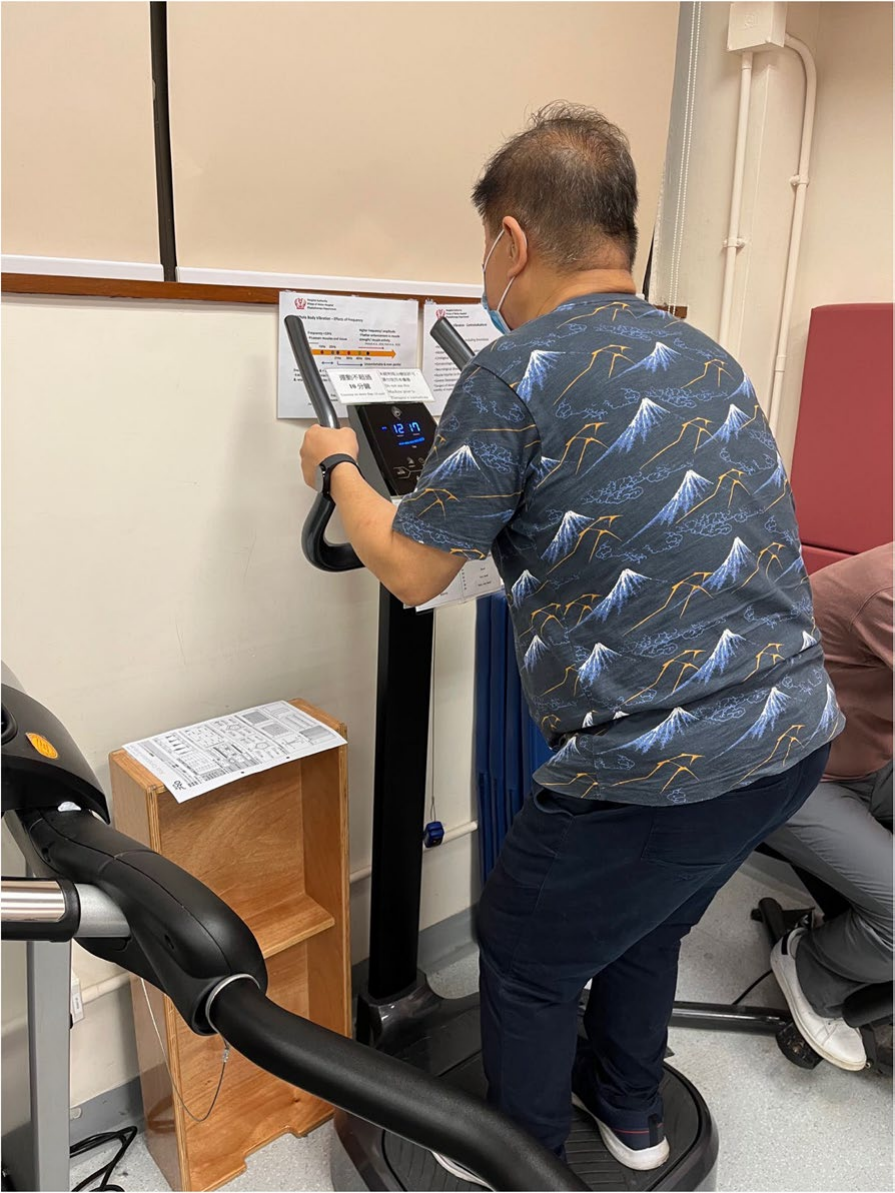
Semi-squatting for knee extensor strengthening using the WBV machine. **a** NPRS. **b** Diseased ROM. **c** Gait (% Column). Knee Society Score. **e** Knee Society Functional Assessment. **f** KOOS. **g** Timed Up and Go. **h** 30-s chair stand. **i** Functional reach

Participants in this group received the same exercise program as the exercise group, with the addition of 10 min of whole-body vibration during semi-squatting or forward lunges (12–16 Hz, varying intensity from low to high depending on patient’s tolerance, using either manual mode with Sonix SW-VH11 (Sonix, Korea)/TurboSonic TT2590-Ovation device (TurboSonic, USA) or auto mode (Leg) with Sonix SW-VH11/ “Training” mode with TurboSonic TT2590-Ovation device; or 35 Hz with either low/high intensity depending on patients’ tolerance using Fitvibexcel Pro Medical device (Fitvibemedical, USA). Forward lunges (90° knee flexion) and semi-squatting (60° knee flexion, weight predominantly through heels) were performed to maximize vibration transmission to the knee (Fig. 4) [33–35].

The intensity of the whole-body vibration intervention was adjusted based on the patient’s tolerance, starting at a lower intensity, and gradually increasing as the patient became accustomed to the sensation. Patients were instructed to maintain proper form during the exercises and to report any discomfort or pain. The physiotherapists closely monitored the patients during the WBV intervention to ensure safety and proper technique.

Control group (*n* = 201).

Participants in the control group did not receive any specific intervention related to the study. However, all participants, including those in the control group, continued to receive regular clinical follow-up and standard medical treatment included regular consultations and prescribed analgesics throughout the study period.

#### Adverse event

No adverse event related to the study interventions was reported in any of the groups.

### Outcome measures

Outcomes were assessed at the baseline, initial and final treatment sessions by licensed physiotherapists who had received specific training in the study protocols and outcome measures.

Primary Outcome Measures are as follows: (1) 11-point numerical pain rating scale (NPRS): Assessed pain in the past week, and (2) Knee injury and Osteoarthritis Outcome Score (KOOS) and subscales, including pain (KOOS-KP), symptoms (KOOS-S), function in daily living (KOOS-ADL), function in sports and recreation (KOOS-Sport/Rec) and knee-related quality of life (KOOS-QOL). Secondary Outcome Measures are as follows: (1) Diseased leg range of motion (ROM) using goniometer, (2) Gait: Normal or limping, (3) Knee Society Score (KSS) and Knee Society Function Assessment (KSFA), and (4) Timed up and go test, 30-s chair stand test, and Functional reach test.

### Sample size calculation

We conducted a pilot study on a small group of patients who received the same exercise program and whole body vibration after receiving ethics approval and before commencement of the actual study. Mean pain levels (in terms of NPRS) and standard deviation (SD) collected between the first session and final session of the program were used to calculate the effect size and the sample size. Given the calculated effect size at 0.50, two-sided α at 0.05 and power at 0.95, the total sample size was 103. Calculations were performed using G*Power 3.1.9.7. Considering the possibility of patient dropout, a mark-up of 20% was applied and the sample size was 124 per group.

### Statistical analysis

Demographic characteristics, Kellgren and Lawrence (KL) grades, pain duration (years), walking tolerance (minutes), and alignment (valgus, varus, neutral) were summarized by mean ± SD for numeric data or N (%) for categorical data where appropriate. NPRS, diseased leg ROM, gait, KSS, KSFA, KOOS and subscales, Timed up and go test, 30-s chair stand test, and functional reach test measured at the first session (1st session) were compared among the 3 groups using ANOVA with *post-hoc* Bonferroni multiple comparison correction. Stepwise linear regression modelling on potential factors, both crude and stepwise controlling for confounding variables, was carried out to eliminate the influence of possible confounding factors. Dummy variables were created for confounding factors in categorical format. Longitudinal comparisons of outcomes and scores in the “Exercise group” and “Exercise + WBV group” were compared using Student’s *t*-test (for numeric variables) or the chi-square test (for categorical variables) where appropriate. Minimal clinically important difference (MCID) of NPRS was calculated to determine the meaningful change of the mean pain score of the patients after coming through the exercise program, using anchor-based method on standard error of mean. All data analyses were carried out using IBM SPSS 28.0 (Armonk, New York, NY, USA). A two-sided *P*-value ≤ 0.05 was considered statistically significant.

## Results

### Demographics and baseline characteristics

The demographics and baseline characteristics of the Control group (*n* = 201), Exercise group (*n* = 227), and Exercise + WBV group (*n* = 127) are presented in Tables 3 and 4. The Exercise + WBV group was significantly younger (mean age 66.86 ± 6.55 years) compared to the Control (70.58 ± 6.22 years) and Exercise (69.40 ± 6.09 years) groups (*P* < 0.001). Gender distribution differed significantly (*P* = 0.002), with more female patients in the Exercise group (75.3%) compared to the Control (59.2%) and Exercise + WBV (67.7%) groups. Knee alignment also varied significantly (*P* = 0.010): valgus alignment was more prevalent in the Exercise group (16.3%) compared to the Control (6.5%) and Exercise + WBV (8.7%) groups.

**Table 3:** Demographics and baseline characteristics in the control, exercise, and exercise + whole-body vibration (WBV) groups

	**Control** **(*n* = 201)**	**Exercise** **(*n* = 227)**	**Exercise + WBV** **(*n* = 127)**	***P-*value**
Age (years)	70.58 ± 6.22	69.40 ± 6.09	66.86 ± 6.55	< 0.001*
Gender, *n* (%)				
Male	82 (45.8) (40.8)	56 (31.3) (24.7)	41 (22.9) (32.3)	0.002*
Female	119 (31.6) (59.2)	171 (45.5) (75.3)	86 (22.9) (67.7)	
Body mass (kg)	68.84 ± 12.40	69.28 ± 12.77	66.53 ± 13.52	0.138
BMI (kg/m^2^)	28.09 ± 4.56	27.81 ± 4.87	26.74 ± 6.06	0.055
Side of affected knee, *n* (%)				
Left knee only	35 (44.3) (17.4)	32 (40.5) (14.1)	12 (15.2) (9.4)	0.080
Right knee only	42 (43.3) (20.9)	37 (38.1) (16.3)	18 (18.6) (14.2)	
Bilateral knee	124 (32.7) (61.7)	158 (41.7) (69.6)	97 (25.6) (76.4)	
KL grading, *n* (%)				
2	20 (43.5) (10.0)	15 (32.6) (6.6)	11 (23.9) (9.4)	0.011*
3	82 (31.2) (40.8)	111 (42.2) (48.9)	70 (26.6) (59.8)	
4	99 (41.9) (49.3)	101 (42.8) (44.5)	36 (15.3) (30.8)	
Pain duration (years)	8.09 ± 3.76	7.60 ± 3.99	6.94 ± 4.04	0.035*
Walking tolerance (min)	30.20 ± 17.36	32.69 ± 17.25	37.95 ± 20.14	< 0.001*
Alignment, *n* (%)				
Valgus	13 (21.3) (6.5)	37 (60.7) (16.3)	11 (18.0) (8.7)	0.010*
Varus	114 (38.6) (56.7)	107 (36.3) (47.1)	74 (25.1) (58.3)	
Neutral	74 (37.2) (36.8)	83 (41.7) (36.6)	42 (21.1) (33.1)	

BMI = Body Mass Index; KL grading = Kellgren and Lawrence grading; WBV = Whole Body Vibration

^*^ Statistical significance (*P* < 0.05)

**Table 4:** Baseline outcomes in the control, exercise, and exercise + whole-body vibration (WBV) groups

	**Control** **(*n* = 201)**	**Exercise** **(*n* = 227)**	**Exercise + WBV (*n* = 127)**	***P-*value**
The 1st session				
NPRS	5.81 ± 1.74^a^	5.35 ± 2.11^a^	5.57 ± 1.82	0.045*
Diseased leg ROM (°)	106.89 ± 18.33	107.89 ± 17.95	110.68 ± 16.52	0.164
Gait, *n* (%)				
Normal	71 (35.0) (35.3)	75 (36.9) (33.0)	57 (28.1) (44.9)	0.077
Limping	130 (36.9) (64.7)	152 (43.2) (67.0)	70 (19.9) (55.1)	
Knee Society Score	60.85 ± 12.55	61.90 ± 19.68	61.20 ± 17.52	0.806
Knee Society Functional Assessment	59.08 ± 13.66^b^	59.13 ± 15.14^c^	63.66 ± 17.12^b,c^	0.012*
KOOS-KP	-	52.24 ± 18.60	54.31 ± 16.95	0.302
KOOS-S	-	49.83 ± 21.86	57.27 ± 19.56	0.002*
KOOS-ADL	-	62.44 ± 20.41	66.99 ± 19.42	0.072
KOOS-Sport/Rec	-	29.93 ± 22.50	38.20 ± 22.93	0.004^*^
KOOS-QOL	-	37.12 ± 21.09	35.96 ± 18.19	0.646
Time and up go	15.23 ± 8.57	14.59 ± 5.70	13.35 ± 6.24	0.060
30-s chair stand	7.57 ± 3.72	8.11 ± 4.39	8.10 ± 3.25	0.298
Functional reach	-	21.61 ± 4.70	23.42 ± 6.51	0.003*

*P*-values after *post-hoc* Bonferroni multiple comparison correction: ^a^ Between Exercise and Control; *P* = 0.039; ^b^ Between Exercise + WBV and Control; *P* = 0.023; ^c^ Between Exercise + WBV and Exercise; *P* = 0.021

NPRS = Numeric Pain Rating Scale; ROM = Range of Motion; WBV = Whole Body Vibration; KOOS = Knee injury and Osteoarthritis Outcome Score; KOOS-KP = Knee injury and Osteoarthritis Outcome score–Knee Pain; KOOS-S = Knee injury and Osteoarthritis Outcome score–Other Symptoms; KOOS-ADL = Knee injury and Osteoarthritis Outcome score–Activities of Daily Living; KOOS-Sport/Rec = Knee injury and Osteoarthritis Outcome score–Sport and Recreation function; KOOS-QOL = Knee injury and Osteoarthritis Outcome score–knee-related Quality of Life

^*^ Statistical significance (*P* < 0.05)

At baseline, the Control group had significantly higher knee pain (mean ± SD; NPRS score 5.81 ± 1.74) compared to the Exercise (5.35 ± 2.11) and Exercise + WBV (5.57 ± 1.82) groups (*P* = 0.045). *post-hoc* Bonferroni correction results showed that Exercise group achieved significant improvement, compared with Control group (*P* = 0.039) (Table 4). The Exercise + WBV group had significantly higher Knee Society Function score (63.66 ± 17.12) compared to the Control group (59.08 ± 13.66) and Exercise group (59.13 ± 15.14) (*P* = 0.012). *post-hoc* Bonferroni correction results showed that Exercise + WBV group attained significant improvement, compared with Control group (*P* = 0.021) and Exercise group (*P* = 0.023). The Exercise + WBV group scored significantly higher on KOOS-S (57.27 ± 19.56) than the Exercise group (49.83 ± 21.86) (*P* = 0.002). The Exercise group had significantly lower scores on the KOOS-Sport/Rec (29.93 ± 22.50) than the Exercise + WBV group (38.20 ± 22.93) (*P* = 0.004). The Exercise + WBV group had significantly higher functional reach score (23.42 ± 6.51) than the Exercise group (21.61 ± 4.70) (*P* = 0.003).

### Longitudinal comparisons between the Exercise group and Exercise + WBV group (baseline vs. 8 weeks)

#### Pain

**Fig. 5:**
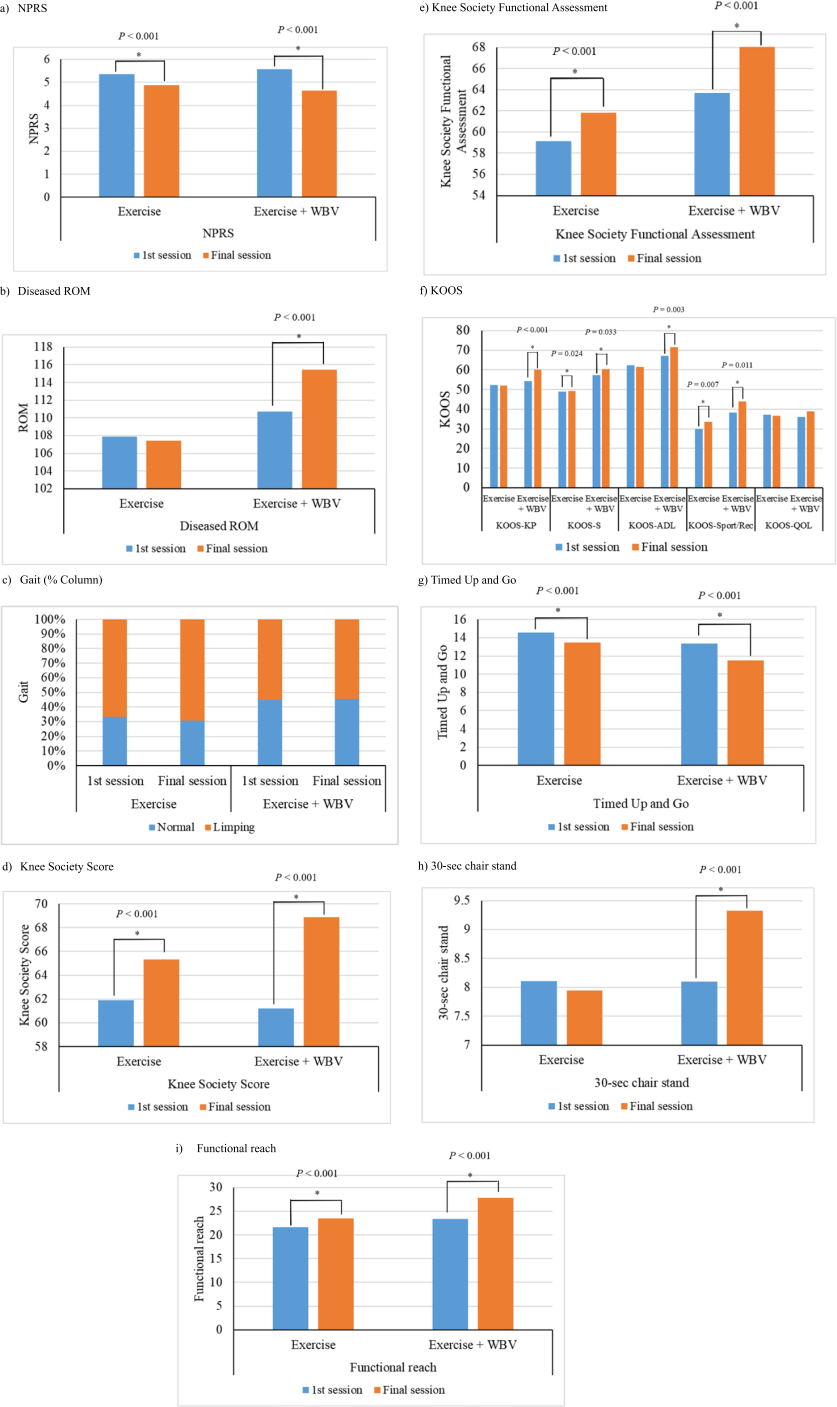
Comparisons of baseline outcomes between Exercise and Exercise + whole-body vibration (WBV) groups

Both exercise modalities significantly reduced knee pain from baseline to the final session (both *P* < 0.001) (Table 5) (Fig. 5). The Exercise + WBV group showed a larger reduction in NPRS score (from 5.57 ± 1.82 to 4.65 ± 2.15) compared to the Exercise group (from 5.35 ± 2.11 to 4.88 ± 1.96). Mean differences between the 1st session and final session were calculated in both groups. Statistically significant differences were found in NPRS (*P* = 0.014), diseased ROM (*P* < 0.001), knee society score (*P* = 0.006), KOOS-KP (*P* < 0.001), KOOS-S (*P* = 0.038), KOOS-ADL (*P* = 0.005) scores, 30-s chair stand (*P* < 0.001), and functional reach (*P* = 0.002).

**Table 5:** Longitudinal comparisons of outcomes and scores among the exercise group and exercise + whole-body vibration (WBV) group

		**The 1st session**	**Final session**	***P* value**	**Difference in mean values between the 1st session and Final session**	***P* value (between Exercise + WBV group and Exercise group)**
*Pain*						
NPRS	Exercise	5.35 ± 2.11	4.88 ± 1.96	< 0.001*	− 0.47 ± 1.59	0.014*
	Exercise + WBV	5.57 ± 1.82	4.65 ± 2.15	< 0.001*	− 0.92 ± 1.78	
*Range of motion and gait*						
Diseased ROM	Exercise	107.89 ± 17.95	107.42 ± 17.36	0.444	− 0.47 ± 9.18	< 0.001*
	Exercise + WBV	110.68 ± 16.52	115.43 ± 18.59	< 0.001*	4.76 ± 11.46	
Gait, N (%)	Exercise					
	Normal	75 (44.4) (33.0)	70 (72.2) (30.8)	0.689	-	-
	Limping	152 (45.9) (67.0)	157 (77.7) (69.2)		-	
	Exercise + WBV					
	Normal	57 (49.6) (44.9)	58 (50.4) (45.7)	0.900	-	-
	Limping	70 (50.4) (55.1)	69 (49.6) (54.3)		-	
*Knee function and quality of life*						
Knee Society Score	Exercise	61.90 ± 19.68	65.34 ± 19.18	< 0.001*	3.44 ± 13.84	0.006*
	Exercise + WBV	61.20 ± 17.52	68.86 ± 17.06	< 0.001*	7.65 ± 13.90	
Knee Society Functional Assessment	Exercise	59.13 ± 15.14	61.82 ± 15.28	< 0.001*	2.69 ± 10.50	0.161
	Exercise + WBV	63.66 ± 17.12	68.07 ± 16.75	< 0.001*	4.41 ± 11.37	
*KOOS*						
KOOS-KP	Exercise	52.24 ± 18.60	51.95 ± 19.38	0.729	− 0.30 ± 12.84	< 0.001*
	Exercise + WBV	54.31 ± 16.95	60.04 ± 17.13	< 0.001*	5.73 ± 14.07	
KOOS-S	Exercise	48.83 ± 21.86	49.25 ± 22.05	0.024*	− 0.58 ± 16.84	0.038*
	Exercise + WBV	57.27 ± 19.56	60.50 ± 18.07	0.033*	3.23 ± 15.94	
KOOS-ADL	Exercise	62.44 ± 20.41	61.56 ± 19.93	0.399	− 0.88 ± 15.56	0.005*
	Exercise + WBV	66.99 ± 19.42	71.52 ± 16.32	0.003^*^	4.53 ± 14.20	
KOOS-Sport/Rec	Exercise	29.94 ± 22.50	33.58 ± 24.04	0.007^*^	3.64 ± 20.05	0.430
	Exercise + WBV	38.20 ± 22.93	43.83 ± 22.96	0.011^*^	5.63 ± 20.36	
KOOS-QOL	Exercise	37.12 ± 21.09	36.67 ± 21.07	0.694	− 0.45 ± 17.33	0.133
	Exercise + WBV	35.96 ± 18.19	38.71 ± 19.72	0.112	2.75 ± 16.16	
*Balance, lower extremity strength and functional reach*						
Timed Up and Go	Exercise	14.59 ± 5.70	13.48 ± 5.52	< 0.001*	− 1.11 ± 4.58	0.146
	Exercise + WBV	13.35 ± 6.24	11.49 ± 3.88	< 0.001*	− 1.87 ± 4.94	
30-s chair stand	Exercise	8.11 ± 4.39	7.94 ± 3.43	0.499	− 0.17 ± 3.82	< 0.001*
	Exercise + WBV	8.10 ± 3.25	9.32 ± 3.64	< 0.001*	1.22 ± 2.84	
Functional reach	Exercise	21.61 ± 4.70	23.46 ± 5.49	< 0.001*	1.85 ± 5.25	0.002*
	Exercise + WBV	23.42 ± 6.51	27.77 ± 7.55	< 0.001*	4.35 ± 7.85	

MCID = Minimal Clinically Important Difference; MDC = Minimal Detectable Change; NPRS = Numeric Pain Rating Scale; ROM = Range of Motion; WBV = Whole Body Vibration; KOOS = Knee injury and Osteoarthritis Outcome Score; KOOS-KP = Knee injury and Osteoarthritis Outcome score – Knee Pain; KOOS-S = Knee injury and Osteoarthritis Outcome score – Other Symptoms; KOOS-ADL = Knee injury and Osteoarthritis Outcome score – Activities of Daily Living; KOOS-Sport/Rec = Knee injury and Osteoarthritis Outcome score – Sport and Recreation function; KOOS-QOL = Knee injury and Osteoarthritis Outcome score – knee-related Quality of Life

^*^ Statistical significance (*P* < 0.05)

#### Knee function and quality of life

After the final session, both groups showed significant improvements in knee function:

- ◦ Knee Society Score: Exercise group from 61.90 ± 19.68 to 65.34 ± 19.18; Exercise + WBV group from 61.20 ± 17.52 to 68.86 ± 17.06 (both *P* < 0.001)
- ◦ Functional Assessment: Exercise group from 59.13 ± 15.14 to 61.82 ± 15.28; Exercise + WBV group from 63.66 ± 17.12 to 68.07 ± 16.75 (both *P* < 0.001)
- ◦ KOOS-S: Exercise group from 48.83 ± 21.86 to 49.25 ± 22.05 (*P* = 0.024); Exercise + WBV group from 57.27 ± 19.56 to 60.50 ± 18.07 (*P* = 0.033)
- ◦ KOOS-Sport/Rec: Exercise group from 29.94 ± 22.50 to 33.58 ± 24.04 (*P* = 0.007); Exercise + WBV group from 38.20 ± 22.93 to 43.83 ± 22.96 (*P* = 0.011)

Significant improvements in other KOOS subscales were only observed in the Exercise + WBV group:

- ◦ KOOS-KP: from 54.31 ± 16.95 to 60.40 ± 17.13 (*P* < 0.001)
- ◦ KOOS-ADL: from 66.99 ± 19.42 to 71.52 ± 16.32 (*P* = 0.003)

No significant difference was observed in the KOOS-QOL score in either group.

#### Range of motion and gait

The Exercise + WBV group accomplished significantly improved knee ROM, from 110.68° ± 16.52° to 115.43° ± 18.59° (*P* < 0.001), while the Exercise group showed no significant change. No statistically significant change was observed in gait in either group.

Balance, lower extremity strength and functional reach.

Significant improvements were observed in:

- ◦ Timed Up and Go test: Exercise group from 14.59 ± 5.70 to 13.48 ± 5.52 s; Exercise + WBV group from 13.35 ± 6.24 to 11.49 ± 3.88 s (both *P* < 0.001)
- ◦ Functional reach test: Exercise group from 21.61 ± 4.70 to 23.46 ± 5.49 cm; Exercise + WBV group from 23.42 ± 6.51 to 27.77 ± 7.55 cm (both *P* < 0.001)
- ◦ 30-sec Chair Stand test: Significant improvement only in the Exercise + WBV group from 8.10 ± 3.25 to 9.32 ± 3.64 stands (*P* < 0.001), while the Exercise group showed no significant change.

### Stepwise linear regression analyses

Statistically significant differences were found in NPRS, diseased ROM, Knee Society Score, KOOS-KP, KOOS-S, KOOS-ADL, 30-s chair stand, and functional reach between Exercise + WBV group and Exercise group (*P* value (between Exercise + WBV group.

and Exercise group) < 0.05 (Table 5), entered the linear regression models. Results of linear regression models were tabulated in Supplementary Information. Except KOOS-S in which no statistically significant difference was found after controlling for age (Model 1.2 in Supplementary Information-Table S5), statistically significant differences could still be found in NPRS, diseased ROM, Knee Society Score, KOOS-KP, KOOS-ADL, 30-s chair stand, and functional reach after controlling for age, gender (ref: female), body mass, BMI, side of affected knee (ref: bilateral), KL grades (ref: Grade 2), pain duration, walking tolerance, and alignment (ref: neutral)(Supplementary Information-Tables S1-S4, S6-S8).

### Minimal clinically important difference (MCID) of NPRS

For pain score (NPRS), the difference in SD was 1.78 and the 95% confidence interval lied between 1.12 and 2.44. These results were in line with previous findings that the use of a 2-point reduction as a standard for significant clinical improvement across interventions [36, 37].

## Discussion

This study aimed to compare the efficacy of exercise therapy alone and with the addition of WBV targeting pain relief and function improvement in end-stage knee OA patients awaiting TKR. The results showed that both interventions led to significant improvements in pain and self-reported physical function. The addition of WBV yielded greater improvements in range of motion, KOOS-KP, and KOOS-ADL scores. These findings are consistent with previous studies showing exercise therapy and WBV to be effective in reducing pain and improving function in early and moderate stage knee OA patients [8, 10, 38, 39].

### Clinical implications

This study provided evidence that supervised exercise therapy can significantly improve outcomes even for patients with end-stage knee OA awaiting joint replacement. Incorporating structured exercised therapy into the routine preoperative management of TKR patients could help alleviate symptoms and improve function during protracted surgical waits [40]. Based on the study findings, an 8-week program consisting of 4–5 sessions, with each session including a 20-min education talk, 30-min of group exercises, and 30 min of individual exercises, appears to be an effective approach.

The addition of WBV to traditional exercise therapy led to significantly greater improvements in several outcomes, suggesting that WBV may be a valuable adjunct to optimize the benefits of exercise therapy. For end-stage knee OA patients, a combined approach starting with WBV as an entry-level intervention before progressing to traditional exercises could potentially achieve greater and more sustainable improvements in pain and function. However, the clinical significance of the observed differences between the “Exercise group” and “Exercise + WBV group” should be carefully considered, as the added cost and complexity of incorporating WBV into rehabilitation programs may not be justified for all patients.

### Further research directions

Future studies should aim to validate the findings of this study and address its limitations. Larger, multi-site randomized controlled trials with sham control groups and longer follow-up periods are needed to confirm the sustained, long-term benefits and generalizability of the interventions. Studies directly comparing the effects of exercise therapy and WBV to other conservative management options, such as manual therapy, bracing, or pain medication, would help determine the relative effectiveness of these interventions and guide clinical decision-making.

Standardizing and optimizing exercise and WBV protocols based on the best available evidence could maximize benefits to patients and support policy changes to increase access to conservative management options during prolonged surgical waits. Additionally, future studies should explore the cost-effectiveness of incorporating WBV into rehabilitation programs and its potential impact on healthcare resource utilization and patient outcomes.

### Study limitations

This study has several limitations that should be considered when interpreting the results. First, the non-randomized allocation of participants to the study groups may have introduced the observed differences between groups. Second, the sample size of the exercise + WBV group was relatively small compared to the other groups, which may limit the generalizability of the findings. Third, the follow-up period was limited to 8 weeks, so the long-term effects of the interventions remain unclear. Fourth, the application of additional inclusion and exclusion criteria specifically for the exercise + whole-body vibration (WBV) group, which may have introduced selection bias. These criteria included the ability to stand independently and a walking tolerance > 15 min, and the rest are stated in Table 1 [19]. Fifth, the intensity of the whole-body vibration intervention was adjusted based on patient’s tolerance, which may have introduced variability in the treatment received. Sixth, severe psychological conditions were already carefully considered at recruitment; however, other psychological conditions, including self-efficacy and motivation, can all impact on individual’s perspectives and involvement in the intervention. Finally, the Exercise + WBV group was significantly younger than the other groups, which may have influenced the results. MCID and MDC values of the two primary outcomes, NPRS and KOOS series, indicated that the comparisons showing statistical significances did not necessarily reflect clinical significances. MCID for pain score was 1.5 [41] and KOOS was around 10 points [42]. These results might have impacted the application of results in a clinical setting. This was the first study to describe the effect of WBV in patients with end-stage knee OA. Data generalizability could be improved when study designs are improved and samples are larger.

Future studies should address these limitations by implementing randomization, using sham interventions, recruiting larger and more balanced sample sizes, extending follow-up periods, using consistent inclusion/exclusion criteria, and standardizing intervention protocols to minimize potential confounding factors.

### Study strengths

Despite its limitations, this study has several notable strengths. It is one of the first to examine the effects of exercise therapy and WBV in patients with end-stage knee OA, a population that has been underrepresented in previous research. The study’s findings provide valuable insights into the potential of these interventions to improve pain, function, and quality of life in patients awaiting TKR, highlighting the importance of optimizing conservative management options during prolonged surgical waits.

Exercise therapy and exercise therapy plus WBV significantly reduced knee pain and improved physical function in end-stage knee OA patients awaiting TKR. The addition of WBV led to further improvements in several outcomes compared to exercise alone, suggesting the WBV may be a useful adjunct to optimize the benefits of exercise therapy. These results contribute to the mounting evidence on the effectiveness of conservative management options for end-stage knee OA and underscore the need for further research to refine and standardize these interventions to maximize patient benefits.

## Conclusions

This study demonstrated that both exercise therapy alone and exercise therapy combined with whole-body vibration (WBV) were effective in reducing pain and improving function in end-stage knee osteoarthritis (OA) patients awaiting total knee replacement (TKR). The addition of WBV to exercise therapy led to greater improvements in several outcomes, suggesting that WBV may be a valuable adjunct to optimize the benefits of exercise therapy. These findings have important implications for patient care and healthcare systems, as incorporating these interventions into the preoperative management of TKR patients could significantly improve patient outcomes, reduce healthcare costs, and optimize resource utilization during prolonged surgical waits.

Further research should focus on addressing the limitations of the current study, confirming the generalizability and long-term sustainability of the findings, and exploring strategies to implement these interventions effectively in clinical practice. By prioritizing conservative management options, such as exercise therapy and WBV, healthcare providers can ensure that patients receive the best possible care while awaiting TKR, ultimately leading to better outcomes and more efficient use of healthcare resources.

## Supplementary Information

Supplementary Material 1. Table S1. Stepwise linear regression analysis of difference in mean NPRS with demographic and baseline characteristics as prognostic factors. Dependent variable: Difference in mean NPRS. Table S2. Stepwise linear regression analysis of difference in mean diseased ROM with demographic and baseline characteristics as prognostic factors. Dependent variable: Difference in mean diseased ROM. Table S3. Stepwise linear regression analysis of difference in mean Knee Society Functional Assessment with demographic and baseline characteristics as prognostic factors. Dependent variable: Difference in mean Knee Society Function Score. Table S4. Stepwise linear regression analysis of difference in mean Knee Society Functional Assessment with demographic and baseline characteristics as prognostic factors. Dependent variable: Difference in mean KOOS-KP. Table S5. Stepwise linear regression analysis of difference in mean Knee Society Functional Assessment with demographic and baseline characteristics as prognostic factors. Dependent variable: Difference in mean KOOS-S. Table S6. Stepwise linear regression analysis of difference in mean Knee Society Functional Assessment with demographic and baseline characteristics as prognostic factors. Dependent variable: Difference in mean KOOS-ADL. Table S7. Stepwise linear regression analysis of difference in mean 30-second chair stand with demographic and baseline characteristics as prognostic factors. Dependent variable: Difference in mean 30-second chair stand. Table S8. Stepwise linear regression analysis of difference in mean Functional reach with demographic and baseline characteristics as prognostic factors. Dependent variable: Difference in mean Functional reach

## Data Availability

The study data used to support the findings of this study are available from the corresponding author upon request.

## Abbreviations

OA: Osteoarthritis
TKR: Total knee replacement
WBV: Whole body vibration
ROM: Range of motion
NPRS: Numeric pain rating scale
KOOS: Knee injury and osteoarthritis outcome score
KOOS-KP: Knee injury and osteoarthritis outcome score–pain
KOOS-S: Knee injury and osteoarthritis outcome score–symptoms
KOOS-ADL: Knee injury and osteoarthritis outcome score–function in daily living
KOOS-Sport/Rec: Knee injury and osteoarthritis outcome score–function in sport and recreation
KOOS-QOL: Knee injury and osteoarthritis outcome score–knee-related quality of life
KSS: Knee society scale
KSFA: Knee society functional assessment

## References

[1] Cui A, Li H, Wang D, Zhong J, Chen Y, Lu H. Global, regional prevalence, incidence and risk factors of knee osteoarthritis in population-based studies. EClinicalMedicine. 2020;29–30: 100587. 34505846 10.1016/j.eclinm.2020.100587 PMC7704420

[2] Al-Taiar A, Al-Sabah R, Elsalawy E, Shehab D, Al-Mahmoud S. Attitudes to knee osteoarthritis and total knee replacement in Arab women: a qualitative study. BMC Res Notes. 2013;6:406. 24107658 10.1186/1756-0500-6-406 PMC3851729

[3] Martel-Pelletier J, Barr AJ, Cicuttini FM, Conaghan PG, Cooper C, Goldring MB, Goldring SR, Jones G, Teichtahl AJ, Pelletier J-P. Osteoarthritis Nature Reviews Disease Primers. 2016;2(1):16072. 27734845 10.1038/nrdp.2016.72

[4] Ackerman IN, Bohensky MA, Zomer E, Tacey M, Gorelik A, Brand CA, de Steiger R. The projected burden of primary total knee and hip replacement for osteoarthritis in Australia to the year 2030. BMC Musculoskelet Disord. 2019;20(1):90. 30797228 10.1186/s12891-019-2411-9 PMC6387488

[5] Bohm ER, Dunbar MJ, Frood JJ, Johnson TM, Morris KA. Rehospitalizations, early revisions, infections, and hospital resource use in the first year after hip and knee arthroplasties. J Arthroplasty. 2012;27(2):232-237.e231. 21752579 10.1016/j.arth.2011.05.004

[6] Safiri S, Kolahi A-A, Smith E, Hill C, Bettampadi D, Mansournia MA, Hoy D, Ashrafi-Asgarabad A, Sepidarkish M, Almasi-Hashiani A, et al. Global, regional and national burden of osteoarthritis 1990–2017: a systematic analysis of the Global Burden of Disease Study 2017. Ann Rheum Dis. 2020;79(6):819–28. 32398285 10.1136/annrheumdis-2019-216515

[7] Ackerman IN, Bennell KL, Osborne RH. Decline in Health-Related Quality of Life reported by more than half of those waiting for joint replacement surgery: a prospective cohort study. BMC Musculoskelet Disord. 2011;12:108. 21605398 10.1186/1471-2474-12-108 PMC3121657

[8] Mather RC 3rd, Hug KT, Orlando LA, Watters TS, Koenig L, Nunley RM, Bolognesi MP. Economic evaluation of access to musculoskeletal care: the case of waiting for total knee arthroplasty. BMC Musculoskelet Disord. 2014;15:22. 24438051 10.1186/1471-2474-15-22 PMC3897923

[9] Fransen M, McConnell S, Harmer AR, Van der Esch M, Simic M, Bennell KL: Exercise for osteoarthritis of the knee. Cochrane Database of Systematic Reviews 2015(1). 10.1002/14651858.CD004376.pub3 PMC10094004 25569281

[10] Uthman OA, van der Windt DA, Jordan JL, Dziedzic KS, Healey EL, Peat GM, Foster NE. Exercise for lower limb osteoarthritis: systematic review incorporating trial sequential analysis and network meta-analysis. BMJ. 2013;347: f5555. 24055922 10.1136/bmj.f5555 PMC3779121

[11] Wallis JA, Webster KE, Levinger P, Taylor NF. What proportion of people with hip and knee osteoarthritis meet physical activity guidelines? A systematic review and meta-analysis. Osteoarthritis Cartilage. 2013;21(11):1648–59. 23948979 10.1016/j.joca.2013.08.003

[12] Wallis JA, Taylor NF. Pre-operative interventions (non-surgical and non-pharmacological) for patients with hip or knee osteoarthritis awaiting joint replacement surgery – a systematic review and meta-analysis. Osteoarthritis Cartilage. 2011;19(12):1381–95. 21959097 10.1016/j.joca.2011.09.001

[13] van Heuvelen MJG, Rittweger J, Judex S, Sañudo B, Seixas A, Fuermaier ABM, Tucha O, Nyakas C, Marín PJ, Taiar R et al.: Reporting Guidelines for Whole-Body Vibration Studies in Humans, Animals and Cell Cultures: A Consensus Statement from an International Group of Experts. Biology (Basel) 2021, 10(10). 10.3390/biology10100965 PMC8533415 34681065

[14] Wuestefeld A, Fuermaier ABM, Bernardo-Filho M, da Cunha de Sá-Caputo D, Rittweger J, Schoenau E, Stark C, Marin PJ, Seixas A, Judex S et al.: Towards reporting guidelines of research using whole-body vibration as training or treatment regimen in human subjects-A Delphi consensus study. PLoS One 2020, 15(7):e0235905. 10.1371/journal.pone.0235905 PMC7375612 32697809

[15] Wang P, Yang X, Yang Y, Yang L, Zhou Y, Liu C, Reinhardt JD, He C. Effects of whole body vibration on pain, stiffness and physical functions in patients with knee osteoarthritis: a systematic review and meta-analysis. Clin Rehabil. 2015;29(10):939–51. 25525066 10.1177/0269215514564895

[16] Li X, Wang XQ, Chen BL, Huang LY, Liu Y. Whole-Body Vibration Exercise for Knee Osteoarthritis: A Systematic Review and Meta-Analysis. Evid Based Complement Alternat Med. 2015;2015: 758147. 26347287 10.1155/2015/758147 PMC4540999

[17] Moreira-Marconi E, Moura-Fernandes MC, Lopes-Souza P, Teixeira-Silva Y, Reis-Silva A, Marchon RM, Guedes-Aguiar EO, Paineiras-Domingos LL, Sá-Caputo DDC, Morel DS, et al. Evaluation of the temperature of posterior lower limbs skin during the whole body vibration measured by infrared thermography: Cross-sectional study analysis using linear mixed effect model. PLoS ONE. 2019;14(3): e0212512. 30865641 10.1371/journal.pone.0212512 PMC6415782

[18] Fransen M, McConnell S, Harmer AR, Van der Esch M, Simic M, Bennell KL. Exercise for osteoarthritis of the knee: a Cochrane systematic review. Br J Sports Med. 2015;49(24):1554–7. 26405113 10.1136/bjsports-2015-095424

[19] Qiu CG, Chui CS, Chow SKH, Cheung WH, Wong RMY: Effects of Whole-Body Vibration Therapy on Knee Osteoarthritis: A Systematic Review and Meta-Analysis of Randomized Controlled Trials. J Rehabil Med 2022, 54:jrm00266. 10.2340/jrm.v54.2032 PMC8963427 35174868

[20] Ribeiro Kütter C, Moreira-Marconi E, Teixeira-Silva Y, Cristina Moura-Fernandes M, Gonçalves de Meirelles A, José dos Santos Pereira M, Chang S, Alexandre Bachur J, Liane Paineiras-Domingos L, Taiar R et al.: Effects of the Whole-Body Vibration and Auriculotherapy on the Functionality of Knee Osteoarthritis Individuals. Applied Sciences 2019, 9(23):5194.

[21] Moura-Fernandes MC, Moreira-Marconi E, de Meirelles AG, Reis-Silva A, de Souza LFF, Lírio Pereira da Silva A, de Oliveira BBM, de Souza Gama MA, de Oliveira ACC, Batouli-Santos D et al.: Acute Effects of Whole-Body Vibration Exercise on Pain Level, Functionality, and Rating of Exertion of Elderly Obese Knee Osteoarthritis Individuals: A Randomized Study. Applied Sciences 2020, 10(17):5870.

[22] Zhang J, Wang R, Zheng Y, Xu J, Wu Y, Wang X. Effect of Whole-Body Vibration Training on Muscle Activation for Individuals with Knee Osteoarthritis. Biomed Res Int. 2021;2021:6671390. 33855078 10.1155/2021/6671390 PMC8019384

[23] Bemben D, Stark C, Taiar R, Bernardo-Filho M. Relevance of Whole-Body Vibration Exercises on Muscle Strength/Power and Bone of Elderly Individuals. Dose Response. 2018;16(4):1559325818813066. 30559636 10.1177/1559325818813066 PMC6291875

[24] Lam FM, Lau RW, Chung RC, Pang MY. The effect of whole body vibration on balance, mobility and falls in older adults: a systematic review and meta-analysis. Maturitas. 2012;72(3):206–13. 22609157 10.1016/j.maturitas.2012.04.009

[25] Tsuji T, Yoon J, Aiba T, Kanamori A, Okura T, Tanaka K. Effects of whole-body vibration exercise on muscular strength and power, functional mobility and self-reported knee function in middle-aged and older Japanese women with knee pain. Knee. 2014;21(6):1088–95. 25153612 10.1016/j.knee.2014.07.015

[26] Seixas A, Sañudo B, Sá-Caputo D, Taiar R, Bernardo-Filho M. Whole-Body Vibration for Individuals with Reconstructed Anterior Cruciate Ligament: A Systematic Review. Biomed Res Int. 2020;2020:7362069. 32462013 10.1155/2020/7362069 PMC7212274

[27] Dionello CF, de Souza PL, Sá-Caputo D, Morel DS, Moreira-Marconi E, Paineiras-Domingos LL, Frederico E, Guedes-Aguiar E, Paiva PC, Taiar R, et al. Do whole body vibration exercises affect lower limbs neuromuscular activity in populations with a medical condition? A systematic review Restor Neurol Neurosci. 2017;35(6):667–81. 29172012 10.3233/RNN-170765

[28] Gränicher P, Stöggl T, Fucentese SF, Adelsberger R, Swanenburg J. Preoperative exercise in patients undergoing total knee arthroplasty: a pilot randomized controlled trial. Arch Physiother. 2020;10:13. 32774889 10.1186/s40945-020-00085-9 PMC7405420

[29] Pendleton A, Arden N, Dougados M, Doherty M, Bannwarth B, Bijlsma JWJ, Cluzeau F, Cooper C, Dieppe PA, Günther K-P, et al. EULAR recommendations for the management of knee osteoarthritis: report of a task force of the Standing Committee for International Clinical Studies Including Therapeutic Trials (ESCISIT). Ann Rheum Dis. 2000;59(12):936–44. 11087696 10.1136/ard.59.12.936 PMC1753053

[30] Saw MM, Kruger-Jakins T, Edries N, Parker R. Significant improvements in pain after a six-week physiotherapist-led exercise and education intervention, in patients with osteoarthritis awaiting arthroplasty, in South Africa: a randomised controlled trial. BMC Musculoskelet Disord. 2016;17:236. 27233479 10.1186/s12891-016-1088-6 PMC4884378

[31] Walsh NE, Mitchell HL, Reeves BC, Hurley MV. Integrated exercise and self-management programmes in osteoarthritis of the hip and knee: a systematic review of effectiveness. Physical Therapy Reviews. 2006;11(4):289–97.

[32] Hurley MV, Mitchell H, Walsh NE. In Osteoarthritis, the Psychosocial Benefits of Exercise Are as Important as Physiological Improvements. Exerc Sport Sci Rev. 2003;31:138–43. 12882480 10.1097/00003677-200307000-00007

[33] Park YG, Kwon BS, Park JW, Cha DY, Nam KY, Sim KB, Chang J, Lee HJ. Therapeutic effect of whole body vibration on chronic knee osteoarthritis. Ann Rehabil Med. 2013;37(4):505–15. 24020031 10.5535/arm.2013.37.4.505 PMC3764345

[34] Petit PD, Pensini M, Tessaro J, Desnuelle C, Legros P, Colson SS. Optimal whole-body vibration settings for muscle strength and power enhancement in human knee extensors. J Electromyogr Kinesiol. 2010;20(6):1186–95. 20801671 10.1016/j.jelekin.2010.08.002

[35] Pollock RD, Woledge RC, Mills KR, Martin FC, Newham DJ. Muscle activity and acceleration during whole body vibration: effect of frequency and amplitude. Clin Biomech (Bristol, Avon). 2010;25(8):840–6. 10.1016/j.clinbiomech.2010.05.004 20541297

[36] Alghadir AH, Anwer S, Iqbal A, Iqbal ZA. Test-retest reliability, validity, and minimum detectable change of visual analog, numerical rating, and verbal rating scales for measurement of osteoarthritic knee pain. J Pain Res. 2018;11:851–6. 29731662 10.2147/JPR.S158847 PMC5927184

[37] Salaffi F, Stancati A, Silvestri CA, Ciapetti A, Grassi W. Minimal clinically important changes in chronic musculoskeletal pain intensity measured on a numerical rating scale. Eur J Pain. 2004;8(4):283–91. 15207508 10.1016/j.ejpain.2003.09.004

[38] Sá-Caputo DC, Paineiras-Domingos LL, Oliveira R, Neves MFT, Brandão A, Marin PJ, Sañudo B, Furness T, Taiar R, Bernardo-Filho M. Acute Effects of Whole-Body Vibration on the Pain Level, Flexibility, and Cardiovascular Responses in Individuals With Metabolic Syndrome. Dose Response. 2018;16(4):1559325818802139. 30305807 10.1177/1559325818802139 PMC6176544

[39] Sá-Caputo D, Paineiras-Domingos LL, Francisca-Santos A, Dos Anjos EM, Reis AS, Neves MFT, Oigman W, Oliveira R, Brandão A, Machado CB, et al. Whole-body vibration improves the functional parameters of individuals with metabolic syndrome: an exploratory study. BMC Endocr Disord. 2019;19(1):6. 30626346 10.1186/s12902-018-0329-0 PMC6325843

[40] Raposo F, Ramos M, Lúcia Cruz A. Effects of exercise on knee osteoarthritis: A systematic review. Musculoskeletal Care. 2021;19(4):399–435. 33666347 10.1002/msc.1538

[41] Kanto K, Lähdeoja T, Paavola M, Aronen P, Järvinen TLN, Jokihaara J, Ardern CL, Karjalainen TV, Taimela S. Minimal important difference and patient acceptable symptom state for pain, Constant-Murley score and Simple Shoulder Test in patients with subacromial pain syndrome. BMC Med Res Methodol. 2021;21(1):45. 33676417 10.1186/s12874-021-01241-w PMC7937213

[42] Culvenor A, Crossley K, Kvist J, McPhail S. Minimal important change values on the Knee injury and Osteoarthritis Outome Score (KOOS) predict future Quality Adjusted Life Years (QALYS) in 3,187 patients following acl reconstruction. Osteoarthritis Cartilage. 2020;28:S381.

