# Supplementary material for: Improving pain, function and quality of life in end-stage knee osteoarthritis: a patient-preference cohort study on whole-body vibration and exercise as bridging therapies for total knee replacement
Source: Arthroplasty. 2025 Mar 10;7:16. doi: 10.1186/s42836-025-00301-6 (PMC11892224; doi:10.1186/s42836-025-00301-6)
Supplement: Supplementary file 1 — Supplementary Material 1. Table S1. Stepwise linear regression analysis of difference in mean NPRS with demographic and baseline characteristics as prognostic factors. Dependent variable: Difference in mean NPRS. Table S2. Stepwise linear regression analysis of difference in mean diseased ROM with demographic and baseline characteristics as prognostic factors. Dependent variable: Difference in mean diseased ROM. Table S3. Stepwise linear regression analysis of difference in mean Knee Society Functional Assessment with demographic and baseline characteristics as prognostic factors. Dependent variable: Difference in mean Knee Society Function Score. Table S4. Stepwise linear regression analysis of difference in mean Knee Society Functional Assessment with demographic and baseline characteristics as prognostic factors. Dependent variable: Difference in mean KOOS-KP. Table S5. Stepwise linear regression analysis of difference in mean Knee Society Functional Assessment with demographic and baseline characteristics as prognostic factors. Dependent variable: Difference in mean KOOS-S. Table S6. Stepwise linear regression analysis of difference in mean Knee Society Functional Assessment with demographic and baseline characteristics as prognostic factors. Dependent variable: Difference in mean KOOS-ADL. Table S7. Stepwise linear regression analysis of difference in mean 30-second chair stand with demographic and baseline characteristics as prognostic factors. Dependent variable: Difference in mean 30-second chair stand. Table S8. Stepwise linear regression analysis of difference in mean Functional reach with demographic and baseline characteristics as prognostic factors. Dependent variable: Difference in mean Functional reach [file 42836_2025_301_MOESM1_ESM.docx]

**Supplementary Materials**

**Table S1.** Stepwise linear regression analysis of difference in mean NPRS with demographic and baseline characteristics as prognostic factors. Dependent variable: Difference in mean NPRS

| Model | Exercise + WBV | Age | Gender (Ref: Female) | Body mass | BMI | Side of affected knee (Ref: Bilateral) | KL grading  (Ref: Grade 2) | Pain duration | Walking tolerance | Alignment (Ref: Neutral) | r^2^ | SE | B (95% CI) | *P* value |
| --- | --- | --- | --- | --- | --- | --- | --- | --- | --- | --- | --- | --- | --- | --- |
| 1.1 (Crude) | 🗸 |  |  |  |  |  |  |  |  |  | 0.021 | 0.19 | -0.51 (-0.89, -0.14) | 0.007* |
| 1.2 | 🗸 |  |  |  |  |  |  |  |  |  | 0.025 | 0.19 | -0.55 (-0.93, -0.18) | 0.004* |
|  |  | 🗸 |  |  |  |  |  |  |  |  |  | 0.01 | -0.02 (-1.29, 2.65) | 0.250 |
| 1.3 | 🗸 |  |  |  |  |  |  |  |  |  | 0.026 | 0.19 | -0.57 (-0.94, -0.19) | 0.004* |
|  |  | 🗸 |  |  |  |  |  |  |  |  |  | 0.01 | -0.02 (-0.05, 0.01) | 0.235 |
|  |  |  | Male |  |  |  |  |  |  |  |  | 0.20 | -0.14 (-0.26, 0.54) | 0.700 |
| 1.4 | 🗸 |  |  |  |  |  |  |  |  |  | 0.027 | 0.20 | -0.59 (-0.97, -0.20) | 0.003* |
|  |  | 🗸 |  |  |  |  |  |  |  |  |  | 0.02 | -0.02 (-0.05, -0.01) | 0.197 |
|  |  |  | Male |  |  |  |  |  |  |  |  | 0.22 | 0.19 (-0.24, 0.62) | 0.389 |
|  |  |  |  | 🗸 |  |  |  |  |  |  |  | 0.01 | -0.00 (-0.02, 0.01) | 0.571 |
| 1.5 | 🗸 |  |  |  |  |  |  |  |  |  | 0.028 | 0.20 | -0.59 (-0.97, -0.20) | 0.003* |
|  |  | 🗸 |  |  |  |  |  |  |  |  |  | 0.02 | -0.02 (-0.05, 0.01) | 0.203 |
|  |  |  | Male |  |  |  |  |  |  |  |  | 0.22 | 0.17 (-0.27, 0.61) | 0.443 |
|  |  |  |  | 🗸 |  |  |  |  |  |  |  | 0.01 | -0.00 (-0.02, 0.02) | 0.769 |
|  |  |  |  |  | 🗸 |  |  |  |  |  |  | 0.02 | -0.01 (-0.05, 0.03) | 0.712 |
| 1.6 | 🗸 |  |  |  |  |  |  |  |  |  | 0.037 | 0.20 | -0.61 (-1.00, -0.23) | 0.002* |
|  |  | 🗸 |  |  |  |  |  |  |  |  |  | 0.02 | -0.02 (-0.05, 0.01) | 0.205 |
|  |  |  | Male |  |  |  |  |  |  |  |  | 0.22 | 0.20 (-0.25, 0.64) | 0.385 |
|  |  |  |  | 🗸 |  |  |  |  |  |  |  | 0.01 | -0.00 (-0.02, 0.02) | 0.740 |
|  |  |  |  |  | 🗸 |  |  |  |  |  |  | 0.02 | -0.01 (0.05, 0.03) | 0.651 |
|  |  |  |  |  |  | Left |  |  |  |  |  | 0.28 | -0.45 (-1.00, 0.10) | 0.111 |
|  |  |  |  |  |  | Right |  |  |  |  |  | 0.25 | 0.11 (-0.38, 0.60) | 0.667 |
| 1.7 | 🗸 |  |  |  |  |  |  |  |  |  | 0.039 | 0.20 | -0.61 (-1.00, -0.22) | 0.002* |
|  |  | 🗸 |  |  |  |  |  |  |  |  |  | 0.02 | -0.02 (-0.05, 0.01) | 0.198 |
|  |  |  | Male |  |  |  |  |  |  |  |  | 0.23 | 0.19 (-0.25, 0.64) | 0.391 |
|  |  |  |  | 🗸 |  |  |  |  |  |  |  | 0.01 | -0.00 (-0.02, 0.02) | 0.740 |
|  |  |  |  |  | 🗸 |  |  |  |  |  |  | 0.02 | -0.01 (-0.05, 0.03) | 0.622 |
|  |  |  |  |  |  | Left |  |  |  |  |  | 0.28 | -0.45 (-1.01, 0.10) | 0.107 |
|  |  |  |  |  |  | Right |  |  |  |  |  | 0.25 | 0.11 (-0.39, 0.60) | 0.676 |
|  |  |  |  |  |  |  | 3 |  |  |  |  | 0.35 | 0.35 (-0.34, 1.04) | 0.320 |
|  |  |  |  |  |  |  | 4 |  |  |  |  | 0.36 | 0.29 (-0.42, 1.01) | 0.423 |
| 1.8 | 🗸 |  |  |  |  |  |  |  |  |  | 0.040 | 0.20 | -0.61 (-1.01, -0.22) | 0.002* |
|  |  | 🗸 |  |  |  |  |  |  |  |  |  | 0.02 | -0.02 (-0.05, 0.01) | 0.211 |
|  |  |  | Male |  |  |  |  |  |  |  |  | 0.23 | 0.20 (-0.25, 0.64) | 0.391 |
|  |  |  |  | 🗸 |  |  |  |  |  |  |  | 0.01 | -0.00 (-0.02, 0.02) | 0.713 |
|  |  |  |  |  | 🗸 |  |  |  |  |  |  | 0.02 | -0.01 (-0.05, 0.03) | 0.654 |
|  |  |  |  |  |  | Left |  |  |  |  |  | 0.28 | -0.46 (-1.01, 0.10) | 0.104 |
|  |  |  |  |  |  | Right |  |  |  |  |  | 0.25 | 0.10 (-0.40, 0.59) | 0.700 |
|  |  |  |  |  |  |  | 3 |  |  |  |  | 0.35 | 0.35 (-0.34, 1.03) | 0.324 |
|  |  |  |  |  |  |  | 4 |  |  |  |  | 0.37 | 0.30 (-0.42, 1.01) | 0.418 |
|  |  |  |  |  |  |  |  | 🗸 |  |  |  | 0.02 | -0.01 (-0.05, 0.04) | 0.761 |
| 1.9 | 🗸 |  |  |  |  |  |  |  |  |  | 0.040 | 0.20 | -0.61 (-1.01, -0.22) | 0.002* |
|  |  | 🗸 |  |  |  |  |  |  |  |  |  | 0.02 | -0.02 (-0.05, 0.01) | 0.209 |
|  |  |  | Male |  |  |  |  |  |  |  |  | 0.23 | 0.19 (-0.25, 0.64) | 0.392 |
|  |  |  |  | 🗸 |  |  |  |  |  |  |  | 0.01 | -0.00 (-0.02, 0.02) | 0.707 |
|  |  |  |  |  | 🗸 |  |  |  |  |  |  | 0.02 | -0.01 (-0.05, 0.03) | 0.647 |
|  |  |  |  |  |  | Left |  |  |  |  |  | 0.28 | -0.46 (-1.01, 0.10) | 0.106 |
|  |  |  |  |  |  | Right |  |  |  |  |  | 0.25 | 0.10 (-0.40, 0.59) | 0.704 |
|  |  |  |  |  |  |  | 3 |  |  |  |  | 0.35 | 0.34 (-0.35, 1.03) | 0.327 |
|  |  |  |  |  |  |  | 4 |  |  |  |  | 0.37 | 0.29 (-0.42, 1.01) | 0.421 |
|  |  |  |  |  |  |  |  | 🗸 |  |  |  | 0.02 | -0.01 (-0.05, 0.04) | 0.770 |
|  |  |  |  |  |  |  |  |  | 🗸 |  |  | 0.01 | -0.00 (-0.01, 0.01) | 0.895 |
| 1.10 | 🗸 |  |  |  |  |  |  |  |  |  | 0.048 | 0.20 | -0.58 (-0.98, -0.19) | 0.004* |
|  |  | 🗸 |  |  |  |  |  |  |  |  |  | 0.02 | -0.02 (-0.05, 0.01) | 0.218 |
|  |  |  | Male |  |  |  |  |  |  |  |  | 0.23 | 0.25 (-0.20, 0.70) | 0.279 |
|  |  |  |  | 🗸 |  |  |  |  |  |  |  | 0.01 | -0.00 (0.02, 0.01) | 0.693 |
|  |  |  |  |  | 🗸 |  |  |  |  |  |  | 0.02 | -0.01 (-0.05, 0.03) | 0.662 |
|  |  |  |  |  |  | Left |  |  |  |  |  | 0.29 | -0.52 (-1.08, 0.05) | 0.071 |
|  |  |  |  |  |  | Right |  |  |  |  |  | 0.25 | 0.07 (-0.42, 0.57) | 0.770 |
|  |  |  |  |  |  |  | 3 |  |  |  |  | 0.35 | 0.34 (-0.35, 1.03) | 0.332 |
|  |  |  |  |  |  |  | 4 |  |  |  |  | 0.37 | 0.33 (-0.39, 1.05) | 0.362 |
|  |  |  |  |  |  |  |  | 🗸 |  |  |  | 0.02 | -0.01 (-0.05, 0.04) | 0.745 |
|  |  |  |  |  |  |  |  |  | 🗸 |  |  | 0.01 | -0.00 (-0.01, 0.01) | 0.890 |
|  |  |  |  |  |  |  |  |  |  | Valgus |  | 0.29 | -0.02 (-0.59, 0.55) | 0.946 |
|  |  |  |  |  |  |  |  |  |  | Varus |  | 0.20 | -0.31 (-0.72, 0.09) | 0.125 |

NPRS: Numeric Pain Rating Scale; WBV: whole-body vibration; BMI: body mass index; KL: Kellgren and Lawrence; SE: Standard error; CI: confidence interval

**Table S2.** Stepwise linear regression analysis of difference in mean diseased ROM with demographic and baseline characteristics as prognostic factors. Dependent variable: Difference in mean diseased ROM

| Model | Exercise + WBV | Age | Gender (Ref: Female) | Body mass | BMI | Side of affected knee (Ref: Bilateral) | KL grading  (Ref: Grade 2) | Pain duration | Walking tolerance | Alignment (Ref: Neutral) | r^2^ | SE | B (95% CI) | P value |
| --- | --- | --- | --- | --- | --- | --- | --- | --- | --- | --- | --- | --- | --- | --- |
| 1.1 (Crude) | 🗸 |  |  |  |  |  |  |  |  |  | 0.060 | 1.16 | 5.42 (3.14, 7.70) | <0.001* |
| 1.2 | 🗸 |  |  |  |  |  |  |  |  |  | 0.068 | 1.17 | 5.77 (3.46, 8.08) | <0.001* |
|  |  | 🗸 |  |  |  |  |  |  |  |  |  | 0.09 | 0.15 (-0.03, 0.32) | 0.10 |
| 1.3 | 🗸 |  |  |  |  |  |  |  |  |  | 0.071 | 1.18 | 5.66 (3.34, 7.97) | <0.001* |
|  |  | 🗸 |  |  |  |  |  |  |  |  |  | 0.09 | 0.14 (-0.03, 0.31) | 0.108 |
|  |  |  | Male |  |  |  |  |  |  |  |  | 1.23 | 1.29 (-1.14, 3.72) | 0.297 |
| 1.4 | 🗸 |  |  |  |  |  |  |  |  |  | 0.072 | 1.20 | 5.75 (3.39, 8.10) | <0.001* |
|  |  | 🗸 |  |  |  |  |  |  |  |  |  | 0.09 | 0.15 (-0.03, 0.33) | 0.096 |
|  |  |  | Male |  |  |  |  |  |  |  |  | 1.34 | 1.06 (-1.57, 3.69) | 0.427 |
|  |  |  |  | 🗸 |  |  |  |  |  |  |  | 0.05 | 0.02 (-0.07, 0.11) | 0.661 |
| 1.5 | 🗸 |  |  |  |  |  |  |  |  |  | 0.076 | 1.20 | 5.75 (3.40, 8.11) | <0.001* |
|  |  | 🗸 |  |  |  |  |  |  |  |  |  | 0.09 | 0.15 (-0.03, 0.33) | 0.107 |
|  |  |  | Male |  |  |  |  |  |  |  |  | 1.37 | 1.40 (-1.28, 4.09) | 0.305 |
|  |  |  |  | 🗸 |  |  |  |  |  |  |  | 0.06 | -0.01 (-0.12, 0.09) | 0.804 |
|  |  |  |  |  | 🗸 |  |  |  |  |  |  | 0.12 | 0.15 (-0.09, 0.38) | 0.228 |
| 1.6 | 🗸 |  |  |  |  |  |  |  |  |  | 0.076 | 1.21 | 5.78 (3.41, 8.16) | <0.001* |
|  |  | 🗸 |  |  |  |  |  |  |  |  |  | 0.09 | 0.15 (-0.03, 0.33) | 0.108 |
|  |  |  | Male |  |  |  |  |  |  |  |  | 1.35 | 1.37 (-1.35, 4.06) | 0.326 |
|  |  |  |  | 🗸 |  |  |  |  |  |  |  | 0.06 | -0.01 (-0.12, 0.10) | 0.805 |
|  |  |  |  |  | 🗸 |  |  |  |  |  |  | 0.12 | 0.15 (-0.09, 0.38) | 0.232 |
|  |  |  |  |  |  | Left |  |  |  |  |  | 1.71 | 0.29 (-3.08, 3.66) | 0.865 |
|  |  |  |  |  |  | Right |  |  |  |  |  | 1.53 | 0.66 (-2.36, 3.67) | 0.669 |
| 1.7 | 🗸 |  |  |  |  |  |  |  |  |  | 0.077 | 1.22 | 5.77 (3.37, 8.17) | <0.001* |
|  |  | 🗸 |  |  |  |  |  |  |  |  |  | 0.09 | 0.15 (-0.03, 0.33) | 0.107 |
|  |  |  | Male |  |  |  |  |  |  |  |  | 1.39 | 1.37 (-1.36, 4.11) | 0.324 |
|  |  |  |  | 🗸 |  |  |  |  |  |  |  | 0.06 | -0.01 (-0.12, 0.10) | 0.812 |
|  |  |  |  |  | 🗸 |  |  |  |  |  |  | 0.12 | 0.15 (-0.09, 0.39) | 0.225 |
|  |  |  |  |  |  | Left |  |  |  |  |  | 1.72 | 0.30 (-3.09, 3.69) | 0.863 |
|  |  |  |  |  |  | Right |  |  |  |  |  | 1.54 | 0.66 (-2.38, 3.68) | 0.671 |
|  |  |  |  |  |  |  | 3 |  |  |  |  | 2.15 | -0.93 (-5.16, 3.29) | 0.663 |
|  |  |  |  |  |  |  | 4 |  |  |  |  | 2.23 | -0.90 (-5.30, 3.49) | 0.687 |
| 1.8 | 🗸 |  |  |  |  |  |  |  |  |  | 0.077 | 1.22 | 5.78 (3.38, 8.18) | <0.001* |
|  |  | 🗸 |  |  |  |  |  |  |  |  |  | 0.09 | 0.15 (-0.04, 0.33) | 0.114 |
|  |  |  | Male |  |  |  |  |  |  |  |  | 1.39 | 1.37 (-1.37, 4.11) | 0.325 |
|  |  |  |  | 🗸 |  |  |  |  |  |  |  | 0.06 | -0.01 (-0.12, 0.10) | 0.840 |
|  |  |  |  |  | 🗸 |  |  |  |  |  |  | 1.24 | 0.15 (-0.10, 0.39) | 0.243 |
|  |  |  |  |  |  | Left |  |  |  |  |  | 1.73 | 0.33 (-3.07, 3.73) | 0.849 |
|  |  |  |  |  |  | Right |  |  |  |  |  | 1.55 | 0.70 (-2.35, 3.74) | 0.654 |
|  |  |  |  |  |  |  | 3 |  |  |  |  | 2.15 | -0.92 (-5.15, 3.31) | 0.668 |
|  |  |  |  |  |  |  | 4 |  |  |  |  | 2.24 | -0.92 (-5.33, 3.48) | 0.680 |
|  |  |  |  |  |  |  |  | 🗸 |  |  |  | 0.14 | 0.04 (-0.24, 0.32) | 0.792 |
| 1.9 | 🗸 |  |  |  |  |  |  |  |  |  | 0.080 | 1.22 | 5.88 (3.47, 8.29) | <0.001* |
|  |  | 🗸 |  |  |  |  |  |  |  |  |  | 0.09 | 0.13 (-0.05, 0.31) | 0.157 |
|  |  |  | Male |  |  |  |  |  |  |  |  | 1.39 | 1.37 (-1.37, 4.10) | 0.327 |
|  |  |  |  | 🗸 |  |  |  |  |  |  |  | 0.06 | -0.02 (-0.13, 0.09) | 0.774 |
|  |  |  |  |  | 🗸 |  |  |  |  |  |  | 0.12 | 0.13 (-0.11, 0.38) | 0.288 |
|  |  |  |  |  |  | Left |  |  |  |  |  | 1.73 | 0.42 (-2.99, 3.82) | 0.810 |
|  |  |  |  |  |  | Right |  |  |  |  |  | 1.55 | 0.63 (-2.42, 3.68) | 0.686 |
|  |  |  |  |  |  |  | 3 |  |  |  |  | 2.15 | -0.98 (-5.21, 3.25) | 0.649 |
|  |  |  |  |  |  |  | 4 |  |  |  |  | 2.24 | -0.99 (-5.40, 3.41) | 0.657 |
|  |  |  |  |  |  |  |  | 🗸 |  |  |  | 0.14 | 0.05 (-0.23, 0.33) | 0.727 |
|  |  |  |  |  |  |  |  |  | 🗸 |  |  | 0.03 | -0.04 (-0.10, 0.03) | 0.262 |
| 1.10 | 🗸 |  |  |  |  |  |  |  |  |  | 0.085 | 1.23 | 5.72 (3.30, 8.14) | <0.001* |
|  |  | 🗸 |  |  |  |  |  |  |  |  |  | 0.09 | 0.13 (-0.05, 0.31) | 0.160 |
|  |  |  | Male |  |  |  |  |  |  |  |  | 1.41 | 1.11 (-1.65, 3.88) | 0.429 |
|  |  |  |  | 🗸 |  |  |  |  |  |  |  | 0.06 | -0.02 (-0.13, 0.09) | 0.776 |
|  |  |  |  |  | 🗸 |  |  |  |  |  |  | 0.13 | 0.13 (-0.11, 0.38) | 0.292 |
|  |  |  |  |  |  | Left |  |  |  |  |  | 1.75 | 0.66 (-2.78, 4.10) | 0.705 |
|  |  |  |  |  |  | Right |  |  |  |  |  | 1.56 | 0.71 (-2.35, 3.77) | 0.648 |
|  |  |  |  |  |  |  | 3 |  |  |  |  | 2.15 | -0.95 (5.19, 3.28) | 0.658 |
|  |  |  |  |  |  |  | 4 |  |  |  |  | 2.25 | -1.16 (-5.58, 3.26) | 0.605 |
|  |  |  |  |  |  |  |  | 🗸 |  |  |  | 0.14 | 0.05 (-0.23, 0.33) | 0.711 |
|  |  |  |  |  |  |  |  |  | 🗸 |  |  | 0.03 | -0.04 (-0.10, 0.03) | 0.262 |
|  |  |  |  |  |  |  |  |  |  | Valgus |  | 1.78 | -0.31 (-3.80, 3.18) | 0.862 |
|  |  |  |  |  |  |  |  |  |  | Varus |  | 1.25 | 1.35 (-1.11, 3.81) | 0.281 |

ROM: range of motion; WBV: whole-body vibration; BMI: body mass index; KL: Kellgren and Lawrence; SE: Standard error; CI: confidence interval

**Table S3.** Stepwise linear regression analysis of difference in mean Knee Society Functional Assessment with demographic and baseline characteristics as prognostic factors. Dependent variable: Difference in mean Knee Society Function Score

| Model | Exercise + WBV | Age | Gender (Ref: Female) | Body mass | BMI | Side of affected knee (Ref: Bilateral) | KL grading  (Ref: Grade 2) | Pain duration | Walking tolerance | Alignment (Ref: Neutral) | r^2^ | SE | B (95% CI) | P value |
| --- | --- | --- | --- | --- | --- | --- | --- | --- | --- | --- | --- | --- | --- | --- |
| 1.1 (Crude) | 🗸 |  |  |  |  |  |  |  |  |  | 0.023 | 1.58 | 4.44 (1.33, 7.54) | 0.005* |
| 1.2 | 🗸 |  |  |  |  |  |  |  |  |  | 0.023 | 1.61 | 4.32 (1.16, 7.48) | 0.008* |
|  |  | 🗸 |  |  |  |  |  |  |  |  |  | 0.12 | -0.05 (-0.29, 0.19) | 0.684 |
| 1.3 | 🗸 |  |  |  |  |  |  |  |  |  | 0.035 | 1.61 | 4.62 (1.46, 7.78) | 0.004* |
|  |  | 🗸 |  |  |  |  |  |  |  |  |  | 0.12 | -0.04 (-0.32, 0.17) | 0.773 |
|  |  |  | Male |  |  |  |  |  |  |  |  | 1.68 | -3.45 (-6.76, -0.14) | 0.051 |
| 1.4 | 🗸 |  |  |  |  |  |  |  |  |  | 0.040 | 1.63 | 4.24 (1.04, 7.44) | 0.010* |
|  |  | 🗸 |  |  |  |  |  |  |  |  |  | 0.12 | -0.08 (-0.32, 0.17) | 0.536 |
|  |  |  | Male |  |  |  |  |  |  |  |  | 1.82 | -2.52 (-6.09, 1.06) | 0.167 |
|  |  |  |  | 🗸 |  |  |  |  |  |  |  | 0.06 | -0.09 (-0.21, 0.04) | 0.182 |
| 1.5 | 🗸 |  |  |  |  |  |  |  |  |  | 0.040 | 1.63 | 4.24 (1.03, 7.45) | 0.010* |
|  |  | 🗸 |  |  |  |  |  |  |  |  |  | 0.12 | -0.08 (-0.32, 0.17) | 0.542 |
|  |  |  | Male |  |  |  |  |  |  |  |  | 1.86 | -2.58 (-6.24, 1.08) | 0.166 |
|  |  |  |  | 🗸 |  |  |  |  |  |  |  | 0.08 | -0.08 (-0.23, 0.07) | 0.292 |
|  |  |  |  |  | 🗸 |  |  |  |  |  |  | 0.16 | -0.03 (-0.35, 0.30) | 0.863 |
| 1.6 | 🗸 |  |  |  |  |  |  |  |  |  | 0.043 | 1.64 | 4.39 (1.16, 7.62) | 0.008* |
|  |  | 🗸 |  |  |  |  |  |  |  |  |  | 0.12 | -0.08 (-0.32, 0.17) | 0.538 |
|  |  |  | Male |  |  |  |  |  |  |  |  | 1.87 | -2.73 (-6.42, 0.95) | 0.146 |
|  |  |  |  | 🗸 |  |  |  |  |  |  |  | 0.08 | -0.08 (-0.22, 0.07) | 0.303 |
|  |  |  |  |  | 🗸 |  |  |  |  |  |  | 0.17 | -0.02 (-0.35, 0.30) | 0.899 |
|  |  |  |  |  |  | Left |  |  |  |  |  | 2.33 | 2.31 (-2.28, 6.90) | 0.322 |
|  |  |  |  |  |  | Right |  |  |  |  |  | 2.09 | 0.01 (-4.10, 4.11) | 0.996 |
| 1.7 | 🗸 |  |  |  |  |  |  |  |  |  | 0.056 | 1.65 | 4.08 (0.84, 7.32) | 0.014* |
|  |  | 🗸 |  |  |  |  |  |  |  |  |  | 0.12 | -0.06 (-0.31, 0.18) | 0.605 |
|  |  |  | Male |  |  |  |  |  |  |  |  | 1.88 | -2.37 (-6.07, 1.33) | 0.208 |
|  |  |  |  | 🗸 |  |  |  |  |  |  |  | 0.08 | -0.07 (-0.22, 0.08) | 0.357 |
|  |  |  |  |  | 🗸 |  |  |  |  |  |  | 0.17 | 0.02 (-0.30, 0.35) | 0.885 |
|  |  |  |  |  |  | Left |  |  |  |  |  | 2.33 | 2.160 (-2.43, 6.74) | 0.356 |
|  |  |  |  |  |  | Right |  |  |  |  |  | 2.08 | -0.13 (-4.23, 3.96) | 0.949 |
|  |  |  |  |  |  |  | 3 |  |  |  |  | 2.90 | -4.60 (-10.31, 1.11) | 0.114 |
|  |  |  |  |  |  |  | 4 |  |  |  |  | 3.02 | -6.33 (-12.27, 0.39) | 0.057 |
| 1.8 | 🗸 |  |  |  |  |  |  |  |  |  | 0.060 | 1.65 | 4.16 (0.92, 7.40) | 0.012* |
|  |  | 🗸 |  |  |  |  |  |  |  |  |  | 0.12 | -0.08 (-0.32, 0.17) | 0.528 |
|  |  |  | Male |  |  |  |  |  |  |  |  | 1.88 | -2.38 (-6.07, 1.32) | 0.207 |
|  |  |  |  | 🗸 |  |  |  |  |  |  |  | 0.08 | -0.06 (-0.21, 0.09) | 0.451 |
|  |  |  |  |  | 🗸 |  |  |  |  |  |  | 0.17 | -0.00 (-0.33, 0.33) | 0.986 |
|  |  |  |  |  |  | Left |  |  |  |  |  | 2.33 | 2.36 (-2.23, 6.95) | 0.313 |
|  |  |  |  |  |  | Right |  |  |  |  |  | 2.09 | 0.13 (-3.99, 4.24) | 0.952 |
|  |  |  |  |  |  |  | 3 |  |  |  |  | 2.90 | -4.52 (-10.22, 1.18) | 0.120 |
|  |  |  |  |  |  |  | 4 |  |  |  |  | 3.02 | -6.46 (-12.40, -0.53) | 0.053 |
|  |  |  |  |  |  |  |  | 🗸 |  |  |  | 0.19 | 0.24 (-0.14, 0.62) | 0.209 |
| 1.9 | 🗸 |  |  |  |  |  |  |  |  |  | 0.064 | 1.65 | 4.30 (1.05, 7.54) | 0.010* |
|  |  | 🗸 |  |  |  |  |  |  |  |  |  | 0.13 | -0.10 (-0.34, 0.15) | 0.435 |
|  |  |  | Male |  |  |  |  |  |  |  |  | 1.88 | -2.38 (-6.08, 1.31) | 0.205 |
|  |  |  |  | 🗸 |  |  |  |  |  |  |  | 0.08 | -0.06 (-0.21, 0.09) | 0.402 |
|  |  |  |  |  | 🗸 |  |  |  |  |  |  | 0.17 | -0.02 (-0.35, 0.31) | 0.903 |
|  |  |  |  |  |  | Left |  |  |  |  |  | 2.34 | 2.48 (-2.12, 7.07) | 0.289 |
|  |  |  |  |  |  | Right |  |  |  |  |  | 2.09 | 0.03 (-4.08, 4.15) | 0.987 |
|  |  |  |  |  |  |  | 3 |  |  |  |  | 2.90 | -4.60 (-10.30, 1.10) | 0.114 |
|  |  |  |  |  |  |  | 4 |  |  |  |  | 3.02 | -6.56 (-12.50, -0.63) | 0.053 |
|  |  |  |  |  |  |  |  | 🗸 |  |  |  | 0.19 | 0.26 (-0.12, 0.64) | 0.180 |
|  |  |  |  |  |  |  |  |  | 🗸 |  |  | 0.04 | -0.05 (-0.13, 0.04) | 0.252 |
| 1.10 | 🗸 |  |  |  |  |  |  |  |  |  | 0.074 | 1.66 | 4.14 (-3.51, 41.23) | 0.013* |
|  |  | 🗸 |  |  |  |  |  |  |  |  |  | 0.13 | -0.11 (-0.35, 0.14) | 0.397 |
|  |  |  | Male |  |  |  |  |  |  |  |  | 1.89 | -2.83 (-6.55, 0.89) | 0.136 |
|  |  |  |  | 🗸 |  |  |  |  |  |  |  | 0.08 | -0.06 (-0.21, 0.09) | 0.444 |
|  |  |  |  |  | 🗸 |  |  |  |  |  |  | 0.17 | -0.03 (-0.36, 0.30) | 0.850 |
|  |  |  |  |  |  | Left |  |  |  |  |  | 2.35 | 3.06 (-1.56, 7.69) | 0.193 |
|  |  |  |  |  |  | Right |  |  |  |  |  | 2.09 | 0.33 (-3.79, 4.44) | 0.876 |
|  |  |  |  |  |  |  | 3 |  |  |  |  | 2.89 | -4.62 (-10.31, 1.07) | 0.111 |
|  |  |  |  |  |  |  | 4 |  |  |  |  | 3.02 | -6.99 (-12.93, -1.05) | 0.051 |
|  |  |  |  |  |  |  |  | 🗸 |  |  |  | 0.19 | 0.27 (-0.11, 0.65) | 0.166 |
|  |  |  |  |  |  |  |  |  | 🗸 |  |  | 0.04 | -0.05 (-0.13, 0.04) | 0.263 |
|  |  |  |  |  |  |  |  |  |  | Valgus |  | 2.39 | 2.43 (-2.27, 7.12) | 0.310 |
|  |  |  |  |  |  |  |  |  |  | Varus |  | 1.68 | 3.17 (-0.14, 6.48) | 0.056 |

WBV: whole-body vibration; BMI: body mass index; KL: Kellgren and Lawrence; SE: Standard error; CI: confidence interval

**Table S4.** Stepwise linear regression analysis of difference in mean Knee Society Functional Assessment with demographic and baseline characteristics as prognostic factors. Dependent variable: Difference in mean KOOS-KP

| Model | Exercise + WBV | Age | Gender (Ref: Female) | Body mass | BMI | Side of affected knee (Ref: Bilateral) | KL grading  (Ref: Grade 2) | Pain duration | Walking tolerance | Alignment (Ref: Neutral) | r^2^ | SE | B (95% CI) | *P*-value |
| --- | --- | --- | --- | --- | --- | --- | --- | --- | --- | --- | --- | --- | --- | --- |
| 1.1 (Crude) | 🗸 |  |  |  |  |  |  |  |  |  | 0.046 | 1.53 | 6.18 (3.17, 9.18) | <0.001* |
| 1.2 | 🗸 |  |  |  |  |  |  |  |  |  | 0.046 | 1.55 | 6.24 (3.18, 9.29) | <0.001* |
|  |  | 🗸 |  |  |  |  |  |  |  |  |  | 0.12 | 0.02 (-0.20, 0.25) | 0.834 |
| 1.3 | 🗸 |  |  |  |  |  |  |  |  |  | 0.049 | 1.56 | 6.40 (3.33, 9.47) | <0.001* |
|  |  | 🗸 |  |  |  |  |  |  |  |  |  | 0.12 | 0.03 (-0.20, 0.26) | 0.783 |
|  |  |  | Male |  |  |  |  |  |  |  |  | 1.63 | -1.85 (-5.06, 1.37) | 0.259 |
| 1.4 | 🗸 |  |  |  |  |  |  |  |  |  | 0.050 | 1.59 | 6.49 (3.37, 9.61) | <0.001* |
|  |  | 🗸 |  |  |  |  |  |  |  |  |  | 0.12 | 0.04 (-0.19, 0.28) | 0.726 |
|  |  |  | Male |  |  |  |  |  |  |  |  | 1.77 | -2.07 (-5.56, 1.41) | 0.242 |
|  |  |  |  | 🗸 |  |  |  |  |  |  |  | 0.06 | 0.02 (-0.10, 0.14) | 0.738 |
| 1.5 | 🗸 |  |  |  |  |  |  |  |  |  | 0.064 | 1.58 | 6.51 (3.40, 9.61) | <0.001* |
|  |  | 🗸 |  |  |  |  |  |  |  |  |  | 0.12 | 0.03 (-0.21, 0.27) | 0.797 |
|  |  |  | Male |  |  |  |  |  |  |  |  | 1.80 | -1.25 (-4.78, 2.29) | 0.489 |
|  |  |  |  | 🗸 |  |  |  |  |  |  |  | 0.07 | -0.06 (-0.21, 0.08) | 0.383 |
|  |  |  |  |  | 🗸 |  |  |  |  |  |  | 0.16 | 0.36 (0.04, 0.67) | 0.056 |
| 1.6 | 🗸 |  |  |  |  |  |  |  |  |  | 0.065 | 1.59 | 6.60 (3.48, 9.73) | <0.001* |
|  |  | 🗸 |  |  |  |  |  |  |  |  |  | 0.12 | 0.03 (-0.21, 0.27) | 0.803 |
|  |  |  | Male |  |  |  |  |  |  |  |  | 1.81 | -1.38 (-4.94, 2.19) | 0.447 |
|  |  |  |  | 🗸 |  |  |  |  |  |  |  | 0.07 | -0.06 (-0.21, 0.08) | 0.387 |
|  |  |  |  |  | 🗸 |  |  |  |  |  |  | 0.16 | 0.36 (-0.04, 0.67) | 0.056 |
|  |  |  |  |  |  | Left |  |  |  |  |  | 2.26 | 1.09 (-3.36, 5.52) | 0.631 |
|  |  |  |  |  |  | Right |  |  |  |  |  | 2.02 | 1.39 (-2.58, 5.36) | 0.492 |
| 1.7 | 🗸 |  |  |  |  |  |  |  |  |  | 0.066 | 1.60 | 6.60 (3.45, 9.76) | <0.001* |
|  |  | 🗸 |  |  |  |  |  |  |  |  |  | 0.12 | 0.03 (-0.21, 0.27) | 0.794 |
|  |  |  | Male |  |  |  |  |  |  |  |  | 1.83 | -1.37 (-4.97, 2.23) | 0.453 |
|  |  |  |  | 🗸 |  |  |  |  |  |  |  | 0.07 | -0.06 (-0.21, 0.08) | 0.390 |
|  |  |  |  |  | 🗸 |  |  |  |  |  |  | 0.16 | 0.36 (-0.04, 0.68) | 0.056 |
|  |  |  |  |  |  | Left |  |  |  |  |  | 2.27 | 1.12-3.35, 5.58) | 0.623 |
|  |  |  |  |  |  | Right |  |  |  |  |  | 2.03 | 1.40 (-2.59, 5.39) | 0.490 |
|  |  |  |  |  |  |  | 3 |  |  |  |  | 2.82 | -1.58 (-7.14, 3.98) | 0.576 |
|  |  |  |  |  |  |  | 4 |  |  |  |  | 2.94 | -1.33 (-7.11, 4.45) | 0.651 |
| 1.8 | 🗸 |  |  |  |  |  |  |  |  |  | 0.066 | 1.61 | 6.61 (3.45, 9.77) | <0.001* |
|  |  | 🗸 |  |  |  |  |  |  |  |  |  | 0.12 | 0.03 (-0.21, 0.27) | 0.802 |
|  |  |  | Male |  |  |  |  |  |  |  |  | 1.83 | -1.37 (-4.98, 2.23) | 0.454 |
|  |  |  |  | 🗸 |  |  |  |  |  |  |  | 0.07 | -0.06 (-0.21, 0.08) | 0.402 |
|  |  |  |  |  | 🗸 |  |  |  |  |  |  | 0.16 | 0.36 (-0.04, 0.68) | 0.058 |
|  |  |  |  |  |  | Left |  |  |  |  |  | 2.28 | 1.13 (-3.35, 0.56) | 0.620 |
|  |  |  |  |  |  | Right |  |  |  |  |  | 2.04 | 1.42 (-2.59, 5.43) | 0.487 |
|  |  |  |  |  |  |  | 3 |  |  |  |  | 2.83 | -1.57 (-7.14, 3.99) | 0.578 |
|  |  |  |  |  |  |  | 4 |  |  |  |  | 2.95 | -1.34 (-7.14, 4.46) | 0.650 |
|  |  |  |  |  |  |  |  | 🗸 |  |  |  | 0.19 | 0.02 (-0.35, 0.39) | 0.923 |
| 1.9 | 🗸 |  |  |  |  |  |  |  |  |  | 0.067 | 1.61 | 6.56 (3.39, 9.74) | <0.001* |
|  |  | 🗸 |  |  |  |  |  |  |  |  |  | 0.12 | 0.04 (-0.20, 0.28) | 0.764 |
|  |  |  | Male |  |  |  |  |  |  |  |  | 1.84 | -1.37 (-4.98, 2.24) | 0.456 |
|  |  |  |  | 🗸 |  |  |  |  |  |  |  | 0.07 | -0.06 (-0.21, 0.09) | 0.420 |
|  |  |  |  |  | 🗸 |  |  |  |  |  |  | 0.16 | 0.37 (0.04, 0.69) | 0.057 |
|  |  |  |  |  |  | Left |  |  |  |  |  | 2.28 | 1.09 (-3.40, 5.58) | 0.633 |
|  |  |  |  |  |  | Right |  |  |  |  |  | 2.04 | 1.45 (-2.57, 5.47) | 0.478 |
|  |  |  |  |  |  |  | 3 |  |  |  |  | 2.83 | -1.55 (-7.12, 4.03) | 0.585 |
|  |  |  |  |  |  |  | 4 |  |  |  |  | 2.95 | -1.31 (-7.11, 4.50) | 0.658 |
|  |  |  |  |  |  |  |  | 🗸 |  |  |  | 0.19 | 0.01 (-0.36, 0.38) | 0.947 |
|  |  |  |  |  |  |  |  |  | 🗸 |  |  | 0.06 | 0.02 (-0.07, 0.10) | 0.702 |
| 1.10 | 🗸 |  |  |  |  |  |  |  |  |  | 0.072 | 1.62 | 6.78 (3.58, 9.97) | <0.001* |
|  |  | 🗸 |  |  |  |  |  |  |  |  |  | 0.12 | 0.03 (-0.21, 0.27) | 0.802 |
|  |  |  | Male |  |  |  |  |  |  |  |  | 1.85 | -1.22 (-4.86, 2.43) | 0.513 |
|  |  |  |  | 🗸 |  |  |  |  |  |  |  | 0.07 | -0.06 (-0.20, 0.09) | 0.459 |
|  |  |  |  |  | 🗸 |  |  |  |  |  |  | 1.16 | 0.36 (0.03, 0.68) | 0.071 |
|  |  |  |  |  |  | Left |  |  |  |  |  | 2.30 | 1.08 (-3.46, 5.61) | 0.641 |
|  |  |  |  |  |  | Right |  |  |  |  |  | 2.05 | 1.53 (-2.51, 5.56) | 0.456 |
|  |  |  |  |  |  |  | 3 |  |  |  |  | 2.84 | -1.62 (-7.20, 3.96) | 0.568 |
|  |  |  |  |  |  |  | 4 |  |  |  |  | 2.96 | -1.32 (-7.15, 4.50) | 0.655 |
|  |  |  |  |  |  |  |  | 🗸 |  |  |  | 0.19 | 0.01 (-0.36, 0.38) | 0.945 |
|  |  |  |  |  |  |  |  |  | 🗸 |  |  | 0.06 | 0.02 (-0.07, 0.10) | 0.682 |
|  |  |  |  |  |  |  |  |  |  | Valgus |  | 2.34 | 2.81 (-1.80, 7.41) | 0.231 |
|  |  |  |  |  |  |  |  |  |  | Varus |  | 1.65 | -0.12 (-3.37, 3.12) | 0.940 |

WBV: whole-body vibration; BMI: body mass index; KL: Kellgren and Lawrence; SE: Standard error; CI: confidence interval

**Table S5.** Stepwise linear regression analysis of difference in mean Knee Society Functional Assessment with demographic and baseline characteristics as prognostic factors. Dependent variable: Difference in mean KOOS-S

| Model | Exercise + WBV | Age | Gender (Ref: Female) | Body mass | BMI | Side of affected knee (Ref: Bilateral) | KL grading  (Ref: Grade 2) | Pain duration | Walking tolerance | Alignment (Ref: Neutral) | r^2^ | SE | B (95% CI) | P value |
| --- | --- | --- | --- | --- | --- | --- | --- | --- | --- | --- | --- | --- | --- | --- |
| 1.1 (Crude) | 🗸 |  |  |  |  |  |  |  |  |  | 0.012 | 1.90 | 3.81 (0.07, 7.55) | 0.046* |
| 1.2 | 🗸 |  |  |  |  |  |  |  |  |  | 0.016 | 1.93 | 3.40 (-0.40, 7.20) | 0.079 |
|  |  | 🗸 |  |  |  |  |  |  |  |  |  | 0.14 | -0.17 (-0.46, 0.11) | 0.234 |
| 1.3 | 🗸 |  |  |  |  |  |  |  |  |  | 0.016 | 1.94 | 3.49 (-0.33, 7.31) | 0.073 |
|  |  | 🗸 |  |  |  |  |  |  |  |  |  | 0.15 | -0.17 (-0.45, 0.12) | 0.247 |
|  |  |  | Male |  |  |  |  |  |  |  |  | 2.03 | -1.00 (-5.00, 3.00) | 0.622 |
| 1.4 | 🗸 |  |  |  |  |  |  |  |  |  | 0.017 | 1.97 | 3.61 (-0.27, 7.49) | 0.068 |
|  |  | 🗸 |  |  |  |  |  |  |  |  |  | 0.15 | -0.15 (-0.45, 0.14) | 0.303 |
|  |  |  | Male |  |  |  |  |  |  |  |  | 2.20 | -1.30 (5.64, 3.03) | 0.554 |
|  |  |  |  | 🗸 |  |  |  |  |  |  |  | 0.08 | 0.03 (-0.13, 0.18) | 0.722 |

WBV: whole-body vibration; BMI: body mass index; KL: Kellgren and Lawrence; SE: Standard error; CI: confidence interval

**Table S6.** Stepwise linear regression analysis of difference in mean Knee Society Functional Assessment with demographic and baseline characteristics as prognostic factors. Dependent variable: Difference in mean KOOS-ADL

| Model | Exercise + WBV | Age | Gender (Ref: Female) | Body mass | BMI | Side of affected knee (Ref: Bilateral) | KL grading  (Ref: Grade 2) | Pain duration | Walking tolerance | Alignment (Ref: Neutral) | r^2^ | SE | B (95% CI) | P value |
| --- | --- | --- | --- | --- | --- | --- | --- | --- | --- | --- | --- | --- | --- | --- |
| 1.1 (Crude) | 🗸 |  |  |  |  |  |  |  |  |  | 0.025 | 1.53 | 6.18 (3.17, 9.18) | 0.005* |
| 1.2 | 🗸 |  |  |  |  |  |  |  |  |  | 0.034 | 1.55 | 6.24 (3.18, 9.29) | 0.008* |
|  |  | 🗸 |  |  |  |  |  |  |  |  |  | 0.12 | 0.02 (-0.20, 0.25) | 0.077 |
| 1.3 | 🗸 |  |  |  |  |  |  |  |  |  | 0.036 | 1.56 | 6.40 (3.33, 9.47) | 0.006* |
|  |  | 🗸 |  |  |  |  |  |  |  |  |  | 0.12 | 0.03 (-0.20, 0.26) | 0.081 |
|  |  |  | Male |  |  |  |  |  |  |  |  | 1.63 | -1.85 (-5.06, 1.37) | 0.515 |
| 1.4 | 🗸 |  |  |  |  |  |  |  |  |  | 0.041 | 1.59 | 6.49 (3.37, 9.61) | 0.004* |
|  |  | 🗸 |  |  |  |  |  |  |  |  |  | 0.12 | 0.04 (-0.19, 0.28) | 0.191 |
|  |  |  | Male |  |  |  |  |  |  |  |  | 1.77 | -2.07 (-5.56, 1.41) | 0.279 |
|  |  |  |  | 🗸 |  |  |  |  |  |  |  | 0.06 | 0.02 (-0.10, 0.14) | 0.198 |
| 1.5 | 🗸 |  |  |  |  |  |  |  |  |  | 0.049 | 1.58 | 6.51 (3.40, 9.61) | 0.005* |
|  |  | 🗸 |  |  |  |  |  |  |  |  |  | 0.12 | 0.03 (-0.21, 0.27) | 0.190 |
|  |  |  | Male |  |  |  |  |  |  |  |  | 1.80 | -1.25 (-4.78, 2.29) | 0.451 |
|  |  |  |  | 🗸 |  |  |  |  |  |  |  | 0.07 | -0.06 (-0.21, 0.08) | 0.698 |
|  |  |  |  |  | 🗸 |  |  |  |  |  |  | 0.16 | 0.36 (0.04, 0.67) | 0.109 |
| 1.6 | 🗸 |  |  |  |  |  |  |  |  |  | 0.053 | 1.59 | 6.60 (3.48, 9.73) | 0.007* |
|  |  | 🗸 |  |  |  |  |  |  |  |  |  | 0.12 | 0.03 (-0.21, 0.27) | 0.201 |
|  |  |  | Male |  |  |  |  |  |  |  |  | 1.81 | -1.38 (-4.94, 2.19) | 0.515 |
|  |  |  |  | 🗸 |  |  |  |  |  |  |  | 0.07 | -0.06 (-0.21, 0.08) | 0.703 |
|  |  |  |  |  | 🗸 |  |  |  |  |  |  | 0.16 | 0.36 (-0.04, 0.67) | 0.119 |
|  |  |  |  |  |  | Left |  |  |  |  |  | 2.26 | 1.09 (-3.36, 5.52) | 0.258 |
|  |  |  |  |  |  | Right |  |  |  |  |  | 2.02 | 1.39 (-2.58, 5.36) | 0.750 |
| 1.7 | 🗸 |  |  |  |  |  |  |  |  |  | 0.058 | 1.60 | 6.60 (3.45, 9.76) | 0.006* |
|  |  | 🗸 |  |  |  |  |  |  |  |  |  | 0.12 | 0.03 (-0.21, 0.27) | 0.183 |
|  |  |  | Male |  |  |  |  |  |  |  |  | 1.83 | -1.37 (-4.97, 2.23) | 0.430 |
|  |  |  |  | 🗸 |  |  |  |  |  |  |  | 0.07 | -0.06 (-0.21, 0.08) | 0.759 |
|  |  |  |  |  | 🗸 |  |  |  |  |  |  | 0.16 | 0.36 (-0.04, 0.68) | 0.166 |
|  |  |  |  |  |  | Left |  |  |  |  |  | 2.27 | 1.12-3.35, 5.58) | 0.285 |
|  |  |  |  |  |  | Right |  |  |  |  |  | 2.03 | 1.40 (-2.59, 5.39) | 0.793 |
|  |  |  |  |  |  |  | 3 |  |  |  |  | 2.82 | -1.58 (-7.14, 3.98) | 0.419 |
|  |  |  |  |  |  |  | 4 |  |  |  |  | 2.94 | -1.33 (-7.11, 4.45) | 0.242 |
| 1.8 | 🗸 |  |  |  |  |  |  |  |  |  | 0.058 | 1.61 | 6.61 (3.45, 9.77) | 0.006* |
|  |  | 🗸 |  |  |  |  |  |  |  |  |  | 0.12 | 0.03 (-0.21, 0.27) | 0.178 |
|  |  |  | Male |  |  |  |  |  |  |  |  | 1.83 | -1.37 (-4.98, 2.23) | 0.434 |
|  |  |  |  | 🗸 |  |  |  |  |  |  |  | 0.07 | -0.06 (-0.21, 0.08) | 0.728 |
|  |  |  |  |  | 🗸 |  |  |  |  |  |  | 0.16 | 0.36 (-0.04, 0.68) | 0.180 |
|  |  |  |  |  |  | Left |  |  |  |  |  | 2.28 | 1.13 (-3.35, 0.56) | 0.294 |
|  |  |  |  |  |  | Right |  |  |  |  |  | 2.04 | 1.42 (-2.59, 5.43) | 0.802 |
|  |  |  |  |  |  |  | 3 |  |  |  |  | 2.83 | -1.57 (-7.14, 3.99) | 0.416 |
|  |  |  |  |  |  |  | 4 |  |  |  |  | 2.95 | -1.34 (-7.14, 4.46) | 0.249 |
|  |  |  |  |  |  |  |  | 🗸 |  |  |  | 0.19 | 0.02 (-0.35, 0.39) | 0.776 |
| 1.9 | 🗸 |  |  |  |  |  |  |  |  |  | 0.058 | 1.61 | 6.56 (3.39, 9.74) | 0.006* |
|  |  | 🗸 |  |  |  |  |  |  |  |  |  | 0.12 | 0.04 (-0.20, 0.28) | 0.175 |
|  |  |  | Male |  |  |  |  |  |  |  |  | 1.84 | -1.37 (-4.98, 2.24) | 0.433 |
|  |  |  |  | 🗸 |  |  |  |  |  |  |  | 0.07 | -0.06 (-0.21, 0.09) | 0.738 |
|  |  |  |  |  | 🗸 |  |  |  |  |  |  | 0.16 | 0.37 (0.04, 0.69) | 0.187 |
|  |  |  |  |  |  | Left |  |  |  |  |  | 2.28 | 1.09 (-3.40, 5.58) | 0.298 |
|  |  |  |  |  |  | Right |  |  |  |  |  | 2.04 | 1.45 (-2.57, 5.47) | 0.792 |
|  |  |  |  |  |  |  | 3 |  |  |  |  | 2.83 | -1.55 (-7.12, 4.03) | 0.417 |
|  |  |  |  |  |  |  | 4 |  |  |  |  | 2.95 | -1.31 (-7.11, 4.50) | 0.250 |
|  |  |  |  |  |  |  |  | 🗸 |  |  |  | 0.19 | 0.01 (-0.36, 0.38) | 0.764 |
|  |  |  |  |  |  |  |  |  | 🗸 |  |  | 0.06 | 0.02 (-0.07, 0.10) | 0.839 |
| 1.10 | 🗸 |  |  |  |  |  |  |  |  |  | 0.066 | 1.62 | 6.78 (3.58, 9.97) | 0.004* |
|  |  | 🗸 |  |  |  |  |  |  |  |  |  | 0.12 | 0.03 (-0.21, 0.27) | 0.158 |
|  |  |  | Male |  |  |  |  |  |  |  |  | 1.85 | -1.22 (-4.86, 2.43) | 0.515 |
|  |  |  |  | 🗸 |  |  |  |  |  |  |  | 0.07 | -0.06 (-0.20, 0.09) | 0.696 |
|  |  |  |  |  | 🗸 |  |  |  |  |  |  | 1.16 | 0.36 (0.03, 0.68) | 0.202 |
|  |  |  |  |  |  | Left |  |  |  |  |  | 2.30 | 1.08 (-3.46, 5.61) | 0.281 |
|  |  |  |  |  |  | Right |  |  |  |  |  | 2.05 | 1.53 (-2.51, 5.56) | 0.826 |
|  |  |  |  |  |  |  | 3 |  |  |  |  | 2.84 | -1.62 (-7.20, 3.96) | 0.430 |
|  |  |  |  |  |  |  | 4 |  |  |  |  | 2.96 | -1.32 (-7.15, 4.50) | 0.240 |
|  |  |  |  |  |  |  |  | 🗸 |  |  |  | 0.19 | 0.01 (-0.36, 0.38) | 0.784 |
|  |  |  |  |  |  |  |  |  | 🗸 |  |  | 0.06 | 0.02 (-0.07, 0.10) | 0.824 |
|  |  |  |  |  |  |  |  |  |  | Valgus |  | 2.34 | 2.81 (-1.80, 7.41) | 0.211 |
|  |  |  |  |  |  |  |  |  |  | Varus |  | 1.65 | -0.12 (-3.37, 3.12) | 0.686 |

WBV: whole-body vibration; BMI: body mass index; KL: Kellgren and Lawrence; SE: Standard error; CI: confidence interval

**Table S7.** Stepwise linear regression analysis of difference in mean 30-second chair stand with demographic and baseline characteristics as prognostic factors. Dependent variable: Difference in mean 30-second chair stand

| Model | Exercise + WBV | Age | Gender (Ref: Female) | Body mass | BMI | Side of affected knee (Ref: Bilateral) | KL grading  (Ref: Grade 2) | Pain duration | Walking tolerance | Alignment (Ref: Neutral) | r^2^ | SE | B (95% CI) | P value |
| --- | --- | --- | --- | --- | --- | --- | --- | --- | --- | --- | --- | --- | --- | --- |
| 1.1 (Crude) | 🗸 |  |  |  |  |  |  |  |  |  | 0.028 | 0.40 | 1.26 (0.47, 2.05) | 0.002* |
| 1.2 | 🗸 |  |  |  |  |  |  |  |  |  | 0.028 | 0.41 | 1.25 (0.45, 2.06) | 0.002* |
|  |  | 🗸 |  |  |  |  |  |  |  |  |  | 0.03 | -0.00 (-0.06, 0.06) | 0.959 |
| 1.3 | 🗸 |  |  |  |  |  |  |  |  |  | 0.032 | 0.41 | 1.21 (0.40, 2.01) | 0.003* |
|  |  | 🗸 |  |  |  |  |  |  |  |  |  | 0.03 | -0.00 (-0.06, 0.06) | 0.902 |
|  |  |  | Male |  |  |  |  |  |  |  |  | 0.43 | 0.53 (-0.32, 1.37) | 0.219 |
| 1.4 | 🗸 |  |  |  |  |  |  |  |  |  | 0.033 | 0.42 | 1.25 (0.43, 2.07) | 0.003* |
|  |  | 🗸 |  |  |  |  |  |  |  |  |  | 0.03 | 0.00 (-0.06, 0.06) | 0.980 |
|  |  |  | Male |  |  |  |  |  |  |  |  | 0.47 | 0.43 (-0.49, 1.34) | 0.359 |
|  |  |  |  | 🗸 |  |  |  |  |  |  |  | 0.02 | 0.01 (-0.02, 0.04) | 0.572 |
| 1.5 | 🗸 |  |  |  |  |  |  |  |  |  | 0.034 | 0.42 | 1.25 (0.43, 2.07) | 0.003* |
|  |  | 🗸 |  |  |  |  |  |  |  |  |  | 0.03 | 0.00 (-0.06, 0.06) | 0.995 |
|  |  |  | Male |  |  |  |  |  |  |  |  | 0.48 | 0.47 (-0.47, 1.41) | 0.325 |
|  |  |  |  | 🗸 |  |  |  |  |  |  |  | 0.02 | 0.01 (-0.03, 0.04) | 0.793 |
|  |  |  |  |  | 🗸 |  |  |  |  |  |  | 0.04 | 0.02 (-0.07, 0.10) | 0.671 |
| 1.6 | 🗸 |  |  |  |  |  |  |  |  |  | 0.041 | 0.42 | 1.19 (0.36, 2.01) | 0.005* |
|  |  | 🗸 |  |  |  |  |  |  |  |  |  | 0.03 | 0.00 (-0.06, 0.06) | 0.984 |
|  |  |  | Male |  |  |  |  |  |  |  |  | 0.48 | 0.55 (-0.39, 1.49) | 0.254 |
|  |  |  |  | 🗸 |  |  |  |  |  |  |  | 0.02 | 0.01 (-0.03, 0.04) | 0.811 |
|  |  |  |  |  | 🗸 |  |  |  |  |  |  | 0.04 | 0.02 (-0.07, 0.10) | 0.699 |
|  |  |  |  |  |  | Left |  |  |  |  |  | 0.60 | -0.83 (-2.00, 0.34) | 0.163 |
|  |  |  |  |  |  | Right |  |  |  |  |  | 0.53 | -0.52 (-1.57, 0.53) | 0.330 |
| 1.7 | 🗸 |  |  |  |  |  |  |  |  |  | 0.047 | 0.42 | 1.11 (0.28, 1.94) | 0.009* |
|  |  | 🗸 |  |  |  |  |  |  |  |  |  | 0.03 | 0.00 (-0.06, 0.07) | 0.933 |
|  |  |  | Male |  |  |  |  |  |  |  |  | 0.48 | 0.63 (-0.32, 1.57) | 0.193 |
|  |  |  |  | 🗸 |  |  |  |  |  |  |  | 0.02 | 0.01 (-0.03, 0.04) | 0.736 |
|  |  |  |  |  | 🗸 |  |  |  |  |  |  | 0.04 | 0.03 (-0.06, 0.11) | 0.564 |
|  |  |  |  |  |  | Left |  |  |  |  |  | 0.60 | -0.89 (-2.06, 0.29) | 0.139 |
|  |  |  |  |  |  | Right |  |  |  |  |  | 0.53 | -0.56 (-1.61, 0.49) | 0.295 |
|  |  |  |  |  |  |  | 3 |  |  |  |  | 0.74 | -0.22 (-1.68, 1.24) | 0.767 |
|  |  |  |  |  |  |  | 4 |  |  |  |  | 0.77 | -0.76 (-2.28, 0.76) | 0.326 |
| 1.8 | 🗸 |  |  |  |  |  |  |  |  |  | 0.054 | 0.42 | 1.09 (0.26, 1.92) | 0.010* |
|  |  | 🗸 |  |  |  |  |  |  |  |  |  | 0.03 | 0.01 (-0.06, 0.07) | 0.819 |
|  |  |  | Male |  |  |  |  |  |  |  |  | 0.48 | 0.63 (-0.32, 1.57) | 0.191 |
|  |  |  |  | 🗸 |  |  |  |  |  |  |  | 0.02 | 0.00 (-0.04, 0.04) | 0.892 |
|  |  |  |  |  | 🗸 |  |  |  |  |  |  | 0.04 | 0.03 (-0.05, 0.12) | 0.439 |
|  |  |  |  |  |  | Left |  |  |  |  |  | 0.60 | -0.95 (-2.13, 0.22) | 0.112 |
|  |  |  |  |  |  | Right |  |  |  |  |  | 0.54 | -0.64 (-1.69, 0.41) | 0.231 |
|  |  |  |  |  |  |  | 3 |  |  |  |  | 0.74 | -0.25 (-1.70, 1.21) | 0.741 |
|  |  |  |  |  |  |  | 4 |  |  |  |  | 0.77 | -0.72 (-2.24, 0.80) | 0.352 |
|  |  |  |  |  |  |  |  | 🗸 |  |  |  | 0.05 | -0.08 -0.17, 0.02) | 0.119 |
| 1.9 | 🗸 |  |  |  |  |  |  |  |  |  | 0.055 | 0.42 | 1.07 (0.24, 1.90) | 0.012* |
|  |  | 🗸 |  |  |  |  |  |  |  |  |  | 0.03 | 0.01 (-0.05, 0.07) | 0.758 |
|  |  |  | Male |  |  |  |  |  |  |  |  | 0.48 | 0.63 (-0.32, 1.58) | 0.191 |
|  |  |  |  | 🗸 |  |  |  |  |  |  |  | 0.02 | 0.00 (-0.04, 0.04) | 0.856 |
|  |  |  |  |  | 🗸 |  |  |  |  |  |  | 0.04 | 0.04 (-0.05, 0.12) | 0.409 |
|  |  |  |  |  |  | Left |  |  |  |  |  | 0.06 | -0.97 (-2.14, 0.21) | 0.107 |
|  |  |  |  |  |  | Right |  |  |  |  |  | 0.54 | -0.63 (-1.68, 0.43) | 0.241 |
|  |  |  |  |  |  |  | 3 |  |  |  |  | 0.74 | -0.23 (-1.70, 1.23) | 0.753 |
|  |  |  |  |  |  |  | 4 |  |  |  |  | 0.77 | -0.71 (-2.23, 0.82) | 0.362 |
|  |  |  |  |  |  |  |  | 🗸 |  |  |  | 0.05 | -0.08 (-0.18, 0.02) | 0.109 |
|  |  |  |  |  |  |  |  |  | 🗸 |  |  | 0.01 | 0.01 (-0.02, 0.03) | 0.542 |
| 1.10 | 🗸 |  |  |  |  |  |  |  |  |  | 0.064 | 0.43 | 1.15 (0.31, 1.98) | 0.007* |
|  |  | 🗸 |  |  |  |  |  |  |  |  |  | 0.03 | 0.01 (-0.06, 0.07) | 0.798 |
|  |  |  | Male |  |  |  |  |  |  |  |  | 0.49 | 0.70 (-0.25, 1.66) | 0.148 |
|  |  |  |  | 🗸 |  |  |  |  |  |  |  | 0.02 | 0.01 (-0.03, 0.04) | 0.802 |
|  |  |  |  |  | 🗸 |  |  |  |  |  |  | 0.04 | 0.03 (-0.05, 0.12) | 0.441 |
|  |  |  |  |  |  | Left |  |  |  |  |  | 0.60 | -1.00 (-2.18, 0.19) | 0.098 |
|  |  |  |  |  |  | Right |  |  |  |  |  | 0.54 | -0.62 (-1.67, 0.44) | 0.252 |
|  |  |  |  |  |  |  | 3 |  |  |  |  | 0.74 | -0.26 (-1.72, 1.20) | 0.728 |
|  |  |  |  |  |  |  | 4 |  |  |  |  | 0.77 | -0.69 (-2.22, 0.83) | 0.372 |
|  |  |  |  |  |  |  |  | 🗸 |  |  |  | 0.05 | -0.08 (-0.18, 0.02) | 0.108 |
|  |  |  |  |  |  |  |  |  | 🗸 |  |  | 0.01 | 0.01 (-0.02, 0.03) | 0.522 |
|  |  |  |  |  |  |  |  |  |  | Valgus |  | 0.61 | 0.85 (-0.35, 2.06) | 0.165 |
|  |  |  |  |  |  |  |  |  |  | Varus |  | 0.43 | -0.18 (-1.03, 0.66) | 0.669 |

WBV: whole-body vibration; BMI: body mass index; KL: Kellgren and Lawrence; SE: Standard error; CI: confidence interval

**Table S8.** Stepwise linear regression analysis of difference in mean Functional reach with demographic and baseline characteristics as prognostic factors. Dependent variable: Difference in mean Functional reach

| Model | Exercise + WBV | Age | Gender (Ref: Female) | Body mass | BMI | Side of affected knee (Ref: Bilateral) | KL grading  (Ref: Grade 2) | Pain duration | Walking tolerance | Alignment (Ref: Neutral) | r^2^ | SE | B (95% CI) | P value |
| --- | --- | --- | --- | --- | --- | --- | --- | --- | --- | --- | --- | --- | --- | --- |
| 1.1 (Crude) | 🗸 |  |  |  |  |  |  |  |  |  | 0.033 | 0.71 | 2.44 (1.04, 3.84) | <0.001* |
| 1.2 | 🗸 |  |  |  |  |  |  |  |  |  | 0.042 | 0.72 | 2.66 (1.24, 4.08) | <0.001* |
|  |  | 🗸 |  |  |  |  |  |  |  |  |  | 0.05 | 0.09 (-0.01, 0.20) | 0.088 |
| 1.3 | 🗸 |  |  |  |  |  |  |  |  |  | 0.042 | 0.73 | 2.63 (1.20, 4.05) | <0.001* |
|  |  | 🗸 |  |  |  |  |  |  |  |  |  | 0.05 | 0.09 (-0.02, 0.20) | 0.10 |
|  |  |  | Male |  |  |  |  |  |  |  |  | 0.76 | 0.39 (-1.10, 1.89) | 0.61 |
| 1.4 | 🗸 |  |  |  |  |  |  |  |  |  | 0.044 | 0.74 | 2.54 (1.09, 3.99) | <0.001* |
|  |  | 🗸 |  |  |  |  |  |  |  |  |  | 0.06 | 0.08 (-0.03, 0.19) | 0.147 |
|  |  |  | Male |  |  |  |  |  |  |  |  | 0.82 | 0.60 (-1.02, 2.22) | 0.467 |
|  |  |  |  | 🗸 |  |  |  |  |  |  |  | 0.03 | -0.02 (-0.08, 0.04) | 0.516 |
| 1.5 | 🗸 |  |  |  |  |  |  |  |  |  | 0.048 | 0.74 | 2.55 (1.10, 4.00) | <0.001* |
|  |  | 🗸 |  |  |  |  |  |  |  |  |  | 0.06 | 0.08 (-0.03, 0.19) | 0.162 |
|  |  |  | Male |  |  |  |  |  |  |  |  | 0.84 | 0.82 (-0.84, 2.47) | 0.332 |
|  |  |  |  | 🗸 |  |  |  |  |  |  |  | 0.03 | -0.04 (-0.11, 0.03) | 0.227 |
|  |  |  |  |  | 🗸 |  |  |  |  |  |  | 0.07 | 0.09 (-0.05, 0.24) | 0.207 |
| 1.6 | 🗸 |  |  |  |  |  |  |  |  |  | 0.049 | 0.74 | 2.50 (1.05, 3.96) | <0.001* |
|  |  | 🗸 |  |  |  |  |  |  |  |  |  | 0.06 | 0.08 (-0.03, 0.19) | 0.162 |
|  |  |  | Male |  |  |  |  |  |  |  |  | 0.85 | 0.87 (-0.79, 2.54) | 0.303 |
|  |  |  |  | 🗸 |  |  |  |  |  |  |  | 0.03 | -0.04 (-0.11, 0.03) | 0.225 |
|  |  |  |  |  | 🗸 |  |  |  |  |  |  | 0.07 | 0.09 (-0.05, 0.24) | 0.212 |
|  |  |  |  |  |  | Left |  |  |  |  |  | 1.05 | -0.52 (-2.59, 1.56) | 0.624 |
|  |  |  |  |  |  | Right |  |  |  |  |  | 0.94 | -0.51 (-2.37, 1.34) | 0.586 |
| 1.7 | 🗸 |  |  |  |  |  |  |  |  |  | 0.054 | 0.75 | 2.60 (1.13, 4.07) | <0.001* |
|  |  | 🗸 |  |  |  |  |  |  |  |  |  | 0.06 | 0.08 (-0.04, 0.19) | 0.182 |
|  |  |  | Male |  |  |  |  |  |  |  |  | 0.85 | 0.76 (-0.92, 2.44) | 0.374 |
|  |  |  |  | 🗸 |  |  |  |  |  |  |  | 0.03 | -0.04 (-0.11, 0.02) | 0.199 |
|  |  |  |  |  | 🗸 |  |  |  |  |  |  | 0.08 | 0.08 (-0.07, 0.23) | 0.287 |
|  |  |  |  |  |  | Left |  |  |  |  |  | 1.06 | -0.46 (-2.54, 1.62) | 0.666 |
|  |  |  |  |  |  | Right |  |  |  |  |  | 0.95 | -0.47 (-2.32, 1.39) | 0.623 |
|  |  |  |  |  |  |  | 3 |  |  |  |  | 1.32 | 0.94 (-1.65, 3.53) | 0.474 |
|  |  |  |  |  |  |  | 4 |  |  |  |  | 1.37 | 1.58 (-1.12, 4.27) | 0.250 |
| 1.8 | 🗸 |  |  |  |  |  |  |  |  |  | 0.054 | 0.75 | 2.61 (1.14, 4.08) | <0.001* |
|  |  | 🗸 |  |  |  |  |  |  |  |  |  | 0.06 | 0.07 (-0.04, 0.19) | 0.191 |
|  |  |  | Male |  |  |  |  |  |  |  |  | 0.85 | 0.76 (-0.92, 2.44) | 0.375 |
|  |  |  |  | 🗸 |  |  |  |  |  |  |  | 0.03 | -0.04 (-0.11, 0.03) | 0.214 |
|  |  |  |  |  | 🗸 |  |  |  |  |  |  | 0.08 | 0.08 (-0.07, 0.23) | 0.306 |
|  |  |  |  |  |  | Left |  |  |  |  |  | 1.06 | -0.44 (-2.53, 1.65) | 0.679 |
|  |  |  |  |  |  | Right |  |  |  |  |  | 0.95 | -0.44 (-2.31, 1.43) | 0.642 |
|  |  |  |  |  |  |  | 3 |  |  |  |  | 1.32 | 0.95 (-1.64, 3.55) | 0.472 |
|  |  |  |  |  |  |  | 4 |  |  |  |  | 1.37 | 1.57 (-1.14, 4.27) | 0.255 |
|  |  |  |  |  |  |  |  | 🗸 |  |  |  | 0.09 | 0.02 (-0.15, 0.19) | 0.816 |
| 1.9 | 🗸 |  |  |  |  |  |  |  |  |  | 0.054 | 0.75 | 2.61 (1.13, 4.09) | <0.001* |
|  |  | 🗸 |  |  |  |  |  |  |  |  |  | 0.06 | 0.08 (-0.04, 0.19) | 0.192 |
|  |  |  | Male |  |  |  |  |  |  |  |  | 0.86 | 0.76 (-0.92, 2.44) | 0.375 |
|  |  |  |  | 🗸 |  |  |  |  |  |  |  | 0.03 | -0.04 (-0.11, 0.03) | 0.219 |
|  |  |  |  |  | 🗸 |  |  |  |  |  |  | 0.08 | 0.08 (-0.07, 0.23) | 0.305 |
|  |  |  |  |  |  | Left |  |  |  |  |  | 1.06 | -0.45 (-2.54, 1.65) | 0.676 |
|  |  |  |  |  |  | Right |  |  |  |  |  | 0.95 | -0.44 (-2.31, 1.44) | 0.645 |
|  |  |  |  |  |  |  | 3 |  |  |  |  | 1.32 | 0.95 (-1.65, 3.55) | 0.471 |
|  |  |  |  |  |  |  | 4 |  |  |  |  | 1.38 | 1.57 (-1.14, 4.28) | 0.255 |
|  |  |  |  |  |  |  |  | 🗸 |  |  |  | 0.09 | 0.02 (-0.15, 0.19) | 0.822 |
|  |  |  |  |  |  |  |  |  | 🗸 |  |  | 0.02 | 0.00 (-0.04, 0.04) | 0.925 |
| 1.10 | 🗸 |  |  |  |  |  |  |  |  |  | 0.055 | 0.76 | 2.59 (1.09, 4.08) | <0.001* |
|  |  | 🗸 |  |  |  |  |  |  |  |  |  | 0.06 | 0.07 (-0.04, 0.19) | 0.200 |
|  |  |  | Male |  |  |  |  |  |  |  |  | 0.87 | 0.70 (-1.00, 2.41) | 0.418 |
|  |  |  |  | 🗸 |  |  |  |  |  |  |  | 0.04 | -0.04 (-0.11, 0.03) | 0.229 |
|  |  |  |  |  | 🗸 |  |  |  |  |  |  | 0.08 | 0.08 (-0.07, 0.23) | 0.316 |
|  |  |  |  |  |  | Left |  |  |  |  |  | 1.08 | -0.37 (-2.49, 1.75) | 0.732 |
|  |  |  |  |  |  | Right |  |  |  |  |  | 0.96 | -0.40 (-2.29, 1.48) | 0.676 |
|  |  |  |  |  |  |  | 3 |  |  |  |  | 1.33 | 0.95 (-1.66, 3.56) | 0.473 |
|  |  |  |  |  |  |  | 4 |  |  |  |  | 1.38 | 1.52 (-1.21, 4.24) | 0.274 |
|  |  |  |  |  |  |  |  | 🗸 |  |  |  | 0.09 | 0.02 (-0.15, 0.19) | 0.813 |
|  |  |  |  |  |  |  |  |  | 🗸 |  |  | 0.02 | 0.00 (-0.04, 0.04) | 0.918 |
|  |  |  |  |  |  |  |  |  |  | Valgus |  | 1.09 | 0.33 (-1.82, 2.48) | 0.761 |
|  |  |  |  |  |  |  |  |  |  | Varus |  | 0.77 | 0.41 (-1.11, 1.93) | 0.595 |

WBV: whole-body vibration; BMI: body mass index; KL: Kellgren and Lawrence; SE: Standard error; CI: confidence interval
